# Remimazolam Ameliorates Autistic‐Like Behaviors via Suppression of Ferroptosis in VTA Dopaminergic Neurons in a Mouse Model of ASD

**DOI:** 10.1002/advs.202508520

**Published:** 2026-02-03

**Authors:** Yuxin Zhang, Jianwei Lin, Chaoyang Tong, Xin Fu, Mengqin Shan, Yue Huang, Kan Zhang, Jijian Zheng

**Affiliations:** ^1^ Department of Anesthesiology Shanghai Children's Medical Center Shanghai Jiao Tong University School of Medicine Shanghai China; ^2^ Department of Oncology (Ward 2) Shanghai Children's Medical Center Shanghai Jiao Tong University School of Medicine Shanghai China; ^3^ Department of Anesthesiology Zhongshan Hospital Fudan University Shanghai China

**Keywords:** autism spectrum disorder, dopaminergic neuron, ferroptosis, remimazolam, ventral tegmental area

## Abstract

Autism spectrum disorder (ASD) is a neurodevelopmental disorder, characterized by social dysfunction and stereotypic behavior, with the neuronal excitation/inhibition (E/I) imbalance as an underlying mechanism. The ultra‐short‐acting GABA‐A receptor agonist remimazolam has elicited unexpected long‐term improvements in agitation and cognition, suggesting a potential role in modulating the E/I imbalance. This study investigates the efficacy and mechanisms of remimazolam on ASD in a valproate (VPA)‐induced model. Intraperitoneal administration of remimazolam significantly ameliorates autistic‐like behaviors, with comparable effects caused by targeted injection of remimazolam into the ventral tegmental area (VTA). Chemogenetic inhibition of VTA dopaminergic neurons in VPA‐exposed mice reverses the therapeutic effects of remimazolam. Conversely, remimazolam also ameliorate the autistic‐like behaviors induced by chemogenetic inhibition. Remimazolam confers protection against VPA‐induced injury to VTA dopaminergic neurons both in vivo and in vitro by inhibiting ferroptosis, as evidenced by the reversal of its key hallmarks: mitochondrial damage, iron overload and lipid peroxidation. Furthermore, ferroptosis agonists reverse the therapeutic effect of remimazolam and ferroptosis inhibitors alleviate the autistic‐like behaviors. This study has demonstrated that remimazolam produces sustained alleviation of autistic‐like behaviors by inhibiting ferroptosis, indicating that ferroptosis may be a critical mechanism to its protection of VTA dopaminergic neurons and resultant behavioral improvement.

## Graphical abstract

The ultra‐short‐acting sedative remimazolam has a sustained therapeutic effect on the core symptoms of VPA‐exposed mice. Remimazolam, a GABA agonist, exerts its therapeutic effects by protecting dopamine neurons in the VTA of VPA‐exposed mice. Meanwhile, ferroptosis is the critical mechanism by which remimazolam protects VTA dopaminergic neurons and alleviates autistic‐like behaviors.

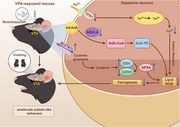

## 1 Introduction

Autism spectrum disorder (ASD) is a type of neurodevelopmental disease with a 1.5%–2% incidence in children and lifelong incurability, characterized by social dysfunction and repetitive behavior as its core symptoms, exerting a significant burden on families and society [1, 2, 3]. The treatment of ASD mainly includes psychotropic drugs and behavioral intervention, but the efficacy is not satisfactory [4, 5, 6]. The alterations of neurotransmitters (Gamma‐Amino Butyric Acid (GABA), dopamine (DA), 5‐hydroxytryptamine (5‐HT), etc.) and the imbalance of neural excitation and inhibition are the most significant pathophysiological changes in ASD, but the specific mechanism is not completely clear [7, 8, 9].

In recent years, studies have found that the brain regions closely related to ASD mainly include the prefrontal cortex (PFC), nucleus accumbens (NAc) and ventral tegmental area (VTA) [10, 11, 12]. Numerous studies have demonstrated that VTA is a crucial brain area involved in controlling social abnormalities in ASD [13, 14, 15]. Dopaminergic neurons account for as high as 60% in VTA and undertake multiple important functions [16]. Although dopaminergic dysfunction has implicated in ASD models, the specific numerical alterations and functions in VTA dopaminergic neurons are not consistently well‐established [17, 18, 19]. In VTA, GABA neurons completely suppress spontaneous firing of DA neurons by activating GABA receptors, in addition to influencing DA neuron activity via local inhibition and long‐range projection [20, 21, 22]. Specifically, the excitability of GABA neurons is enhanced, inhibiting VTA DA neurons and reducing dopamine release [10, 11]. Therefore, direct or indirect regulation of VTA dopaminergic neurons may treat ASD by restoring the functioning of the dopamine neurotransmitter system.

Despite the compelling hypothesis that rectifying the excitation‐inhibition (E/I) balance, potentially via modulation of GABAergic neurotransmission, can represent a key strategy for ameliorating autistic‐like behaviors, a reliable method for its therapeutic application and mechanism is lacking [7, 23, 24]. Remimazolam is an ultra‐short‐acting GABA agonist has the characteristics of fast acting and fast metabolism, with less effect on hemodynamics and respiration [25, 26, 27]. Beyond its established ultra‐short‐acting sedative properties, emerging evidence demonstrates that remimazolam can produce other longer‐acting protective effects, such as alleviation of cognitive dysfunction and agitation [28, 29, 30]. However, no study has investigated the impact of remimazolam on ASD so far. Therefore, we sought to evaluate its therapeutic potential and elucidate the underlying mechanism.

Although the number alteration of VTA dopaminergic neurons across ASD modes is heterogeneous, a consistent decrease is observed in the majority of valproate (VPA)‐induced mouse models [17, 31, 32]. The causes of dopamine neuron depletion may include developmental loss and neuronal damage. Due to the high iron ion environment of the midbrain and the oxidative susceptibility of DA neurons, ferroptosis may be an important mode of death in the reduction of VTA DA neurons [33, 34]. Several studies suggest that ferroptosis may be a potential mechanism and indicator of ASD from database analysis [35, 36, 37]. Meanwhile, some studies on the treatment of ASD with traditional Chinese medicines such as puerarin and Ligustilide have shown therapeutic effects by inhibiting ferroptosis [38, 39, 40]. Although the relationship between remimazolam and ferroptosis is not clear, based on the above analysis, we speculated that remimazolam might play a role in protecting dopaminergic neurons against ferroptosis in ASD.

This study hypothesized that remimazolam alleviates autistic‐like behaviors and exerts its therapeutic effect by modulating the VTA dopaminergic neurons through the suppression of ferroptosis. Our study aims to provide new insights into the development of novel drugs and therapeutic targets for ASD.

## 2 Results

### 2.1 Remimazolam Alleviates Autistic‐Like Behaviors of VPA‐Exposed Mice

**FIGURE 1:**
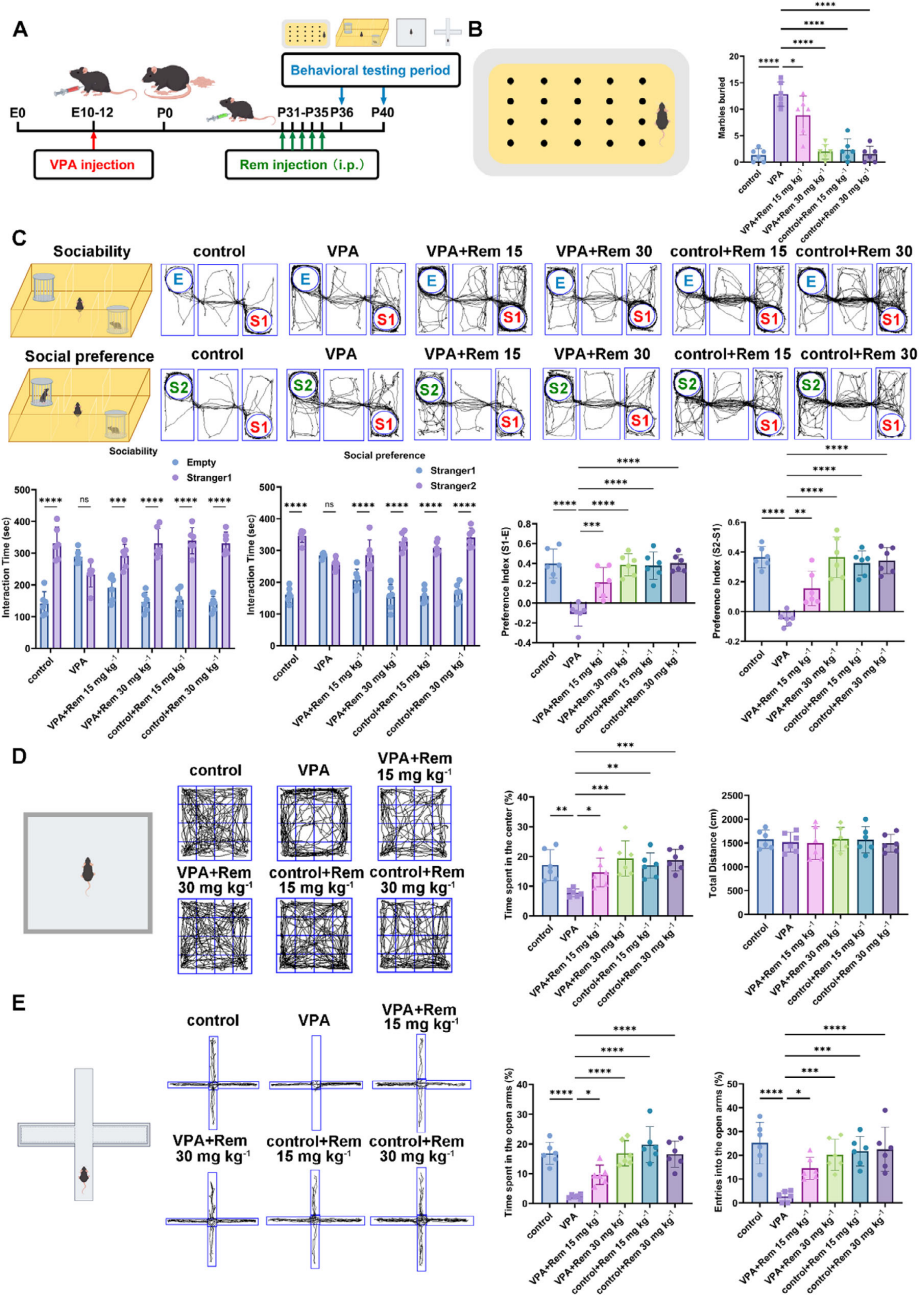
Administration of remimazolam alleviates autism‐related symptoms in VPA‐exposed mice. (A) An outline of the experimental process for administering drugs and performing behavior measures. (B) The schematic of marble burial experiment and the statistical graph of buried marbles number. *n* = 6 per group (C) Representative traces of three‐chamber social interaction test (E: Empty, S1: stranger 1, S2: stranger 2). Statistical graph of sociability and social novelty test. *n* = 6 per group. (D) Representative traces of open‐field test. Statistical graph of the percent of time spent in the center and total distance. *n* = 6 per group. (E) Representative traces of elevated plus maze test. Statistical graph of time and entries spent in open arms. *n* = 6 per group. Values was presented as mean ± SEM and analyzed by one‐way ANOVA or Two‐way ANOVA. ns > 0.05, ^*^ *p* < 0.05, ^**^ *p* < 0.01, ^***^ *p* < 0.001 and ^****^ *p* < 0.0001.

From P31 to P35, both ASD and control mice were treated with daily intraperitoneal injection of remimazolam at doses of 15 or 30 mg kg^−^^1^. Behavioral assessments were conducted following the last injection (Figure 1A). Remimazolam reduced stereotyped behavior in a dose‐dependent manner, with 30 mg kg^−^^1^ producing a more pronounced therapeutic effect than 15 mg kg^−^^1^. Furthermore, the drug itself did not induce stereotypic behavior (Figure 1B). In the three‐chamber social experiment, remimazolam ameliorated the deficits in sociability and social preference observed in VPA‐exposed mice, with a more pronounced effect at 30 mg kg^−^^1^ and did not independently increase social activity (Figure 1C). In the open field experiment, VPA‐exposed mice exhibited anxiety‐like behavior, characterized by a significant avoidance of the center zone. Remimazolam reduced anxiety‐like behavior in VPA‐exposed mice, with 30 mg kg^−^^1^ producing a more pronounced therapeutic effect than 15 mg kg^−^^1^ (Figure 1D). VPA‐exposed mice exhibited pronounced anxiety‐like behavior in the elevated plus maze, as evidenced by a refusal to enter the open arms. Administration of 30 mg kg^−^^1^ remimazolam, better than 15 mg kg^−^^1^, significantly improved this performance (Figure 1E). Therefore, the 30 mg kg^−^^1^ dose of remimazolam was selected for further investigation due to its consistent and robust performance in alleviating core behavioral deficits.

**FIGURE 2:**
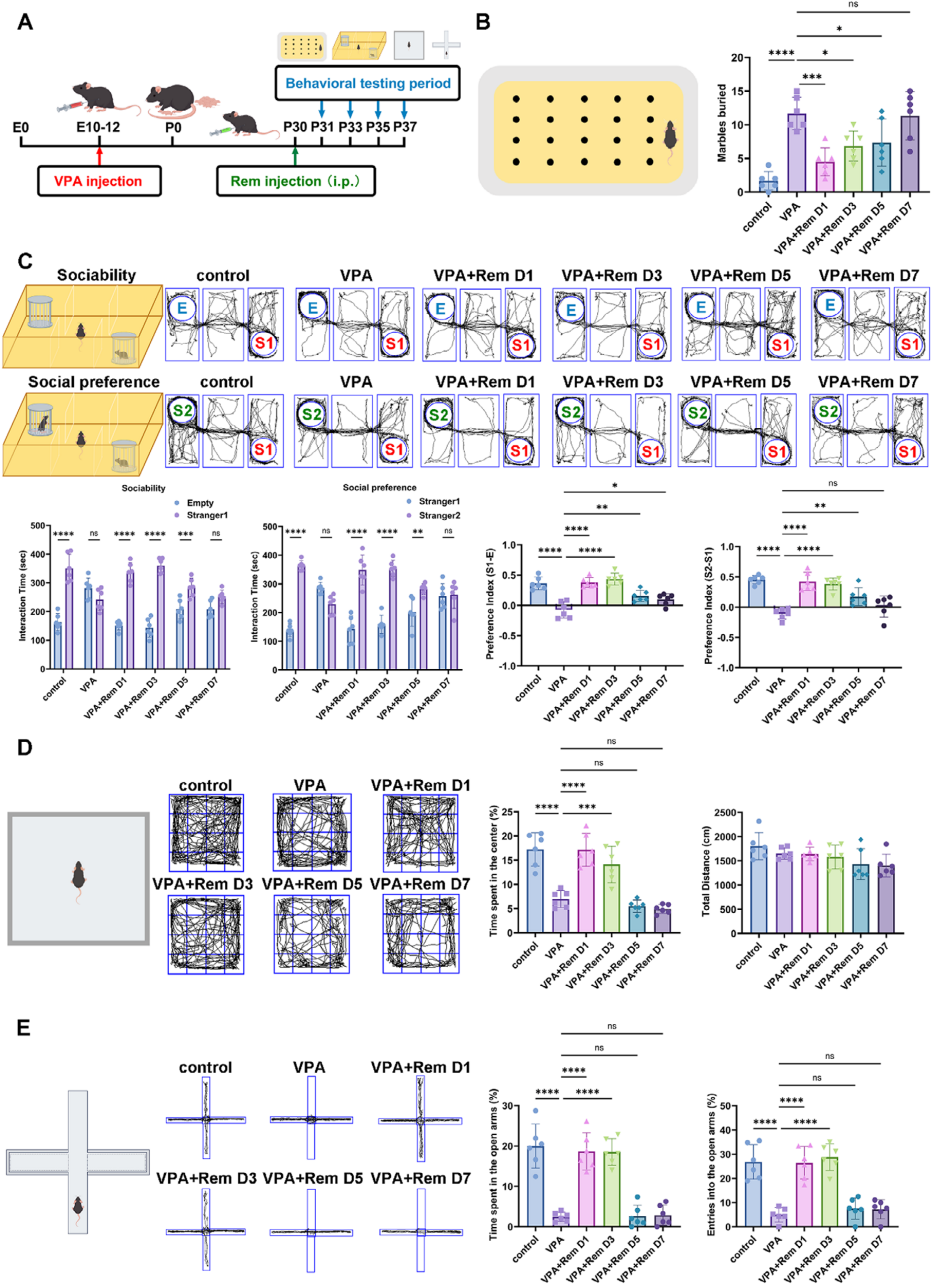
The efficacy and duration of single intraperitoneal injection of remimazolam in VPA‐exposed mice. (A) An outline of the experimental process for administering drugs and performing behavior measures. (B) The schematic of marble burial experiment and the statistical graph of buried marbles number. *n* = 6 per group. (C) Representative traces of three‐chamber social interaction test (E: Empty, S1: stranger 1, S2: stranger 2). Statistical graph of sociability and social novelty test. *n* = 6 per group (D) Representative traces of open‐field test. Statistical graph of the percent of time spent in the center and total distance. *n* = 6 per group. (E) Representative traces of elevated plus maze test. Statistical graph of time and entries spent in open arms. *n* = 6 per group. Values was presented as mean ± SEM and analyzed by one‐way ANOVA or Two‐way ANOVA. ns > 0.05, ^*^ *p* < 0.05, ^**^ *p* < 0.01, ^***^ *p* < 0.001 and ^****^ *p* < 0.0001.

To further investigate the therapeutic effect of remimazolam on VPA‐exposed mice, we performed a single intraperitoneal injection of remimazolam followed by behavioral evaluation every other day to track the duration of its therapeutic effect (Figure 2A). After a single injection of remimazolam, VPA‐exposed mice showed a significant reduction in buried marble, and the therapeutic effect remained statistically decrease until the fifth day after injection (Figure 2B). Compared to social preference, remimazolam's improvement in social novelty persisted longer. While social preference improvement lasted roughly 5 days, social novelty improvement lasted roughly seven days (Figure 2C). The persistent therapeutic effect of remimazolam in open field and elevated plus maze is relatively short, lasting approximately 3 days (Figure 2D,E). Overall, a single intraperitoneal injection of remimazolam could maintain the therapeutic effect for 3–7 days, and the therapeutic effect and duration might range depending on the autism‐related symptoms.

### 2.2 Remimazolam Ameliorates VTA Dopaminergic Neuron Damage in VPA‐Exposed Mice

**FIGURE 3:**
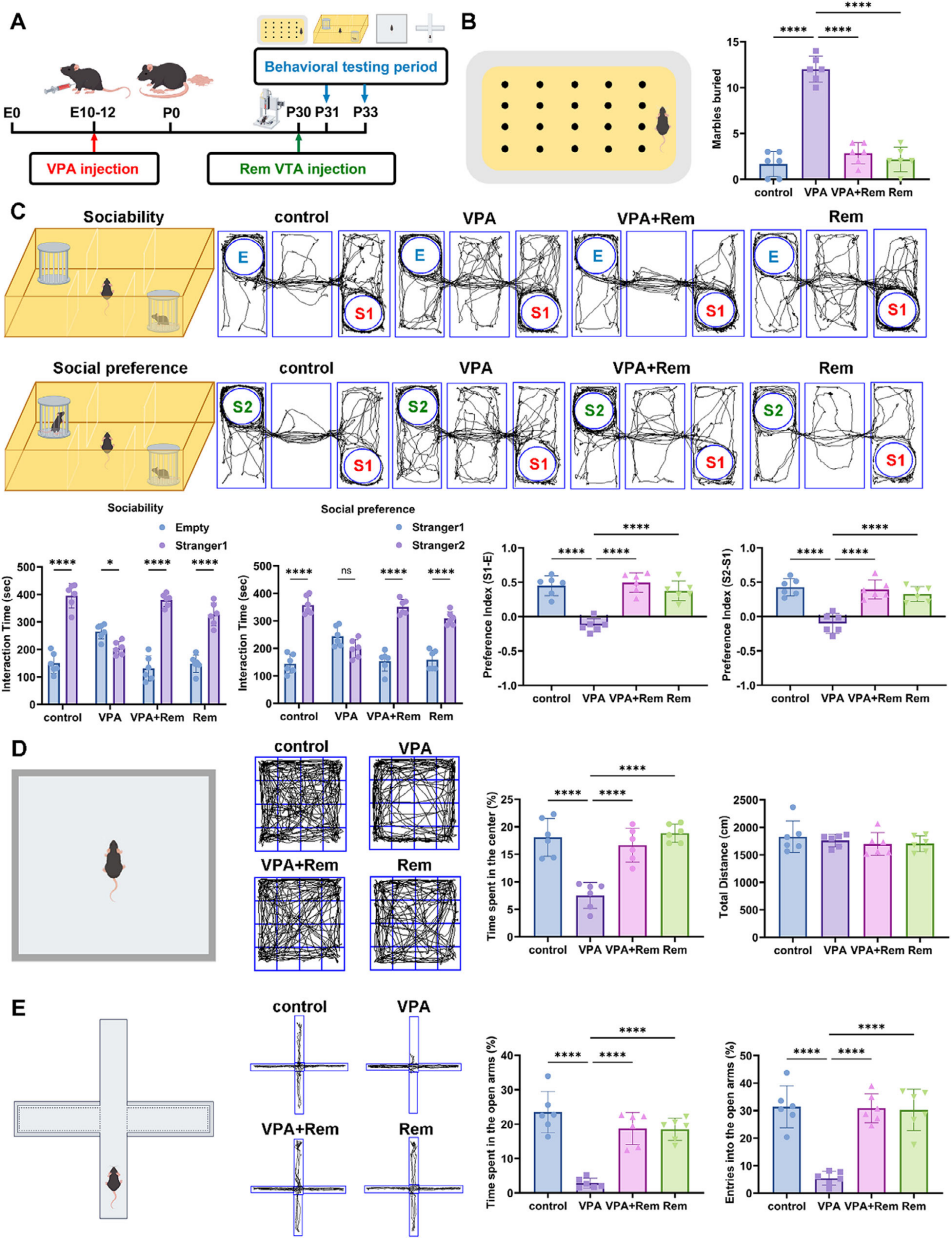
Remimazolam targeted VTA injection improves autism‐like behavior in VPA‐exposed mice. (A) An outline of the experimental process for administering drugs and performing behavior measures. (B) The schematic of marble burial experiment and the statistical graph of buried marbles number. *n* = 6 per group. (C) Representative traces of three‐chamber social interaction test (E: Empty, S1: stranger 1, S2: stranger 2). Statistical graph of sociability and social novelty test. *n* = 6 per group (D) Representative traces of open‐field test. Statistical graph of the percent of time spent in the center and total distance. *n* = 6 per group. (E) Representative traces of elevated plus maze test. Statistical graph of time and entries spent in open arms. *n* = 6 per group. Values was presented as mean ± SEM and analyzed by one‐way ANOVA or Two‐way ANOVA. ns > 0.05, ^*^ *p* < 0.05, ^**^ *p* < 0.01, ^***^ *p* < 0.001 and ^****^ *p* < 0.0001.

To further demonstrate that remimazolam exerts therapeutic effects through the VTA brain region, we conducted targeted injection therapy into the VTA brain region of VPA‐exposed mice and performed behavioral tests (Figure 3A). After targeted injection of remimazolam into the VTA brain area, marble burial was significantly decreased (Figure 3B). Remimazolam has no additional impact on itself. In the three‐chamber social experiment, the significant therapeutic effect of remimazolam was also observed (Figure 3C). The open field and elevated plus maze trials demonstrated positive therapeutic benefits after injection of remimazolam in VTA (Figure 3D,E).

**FIGURE 4:**
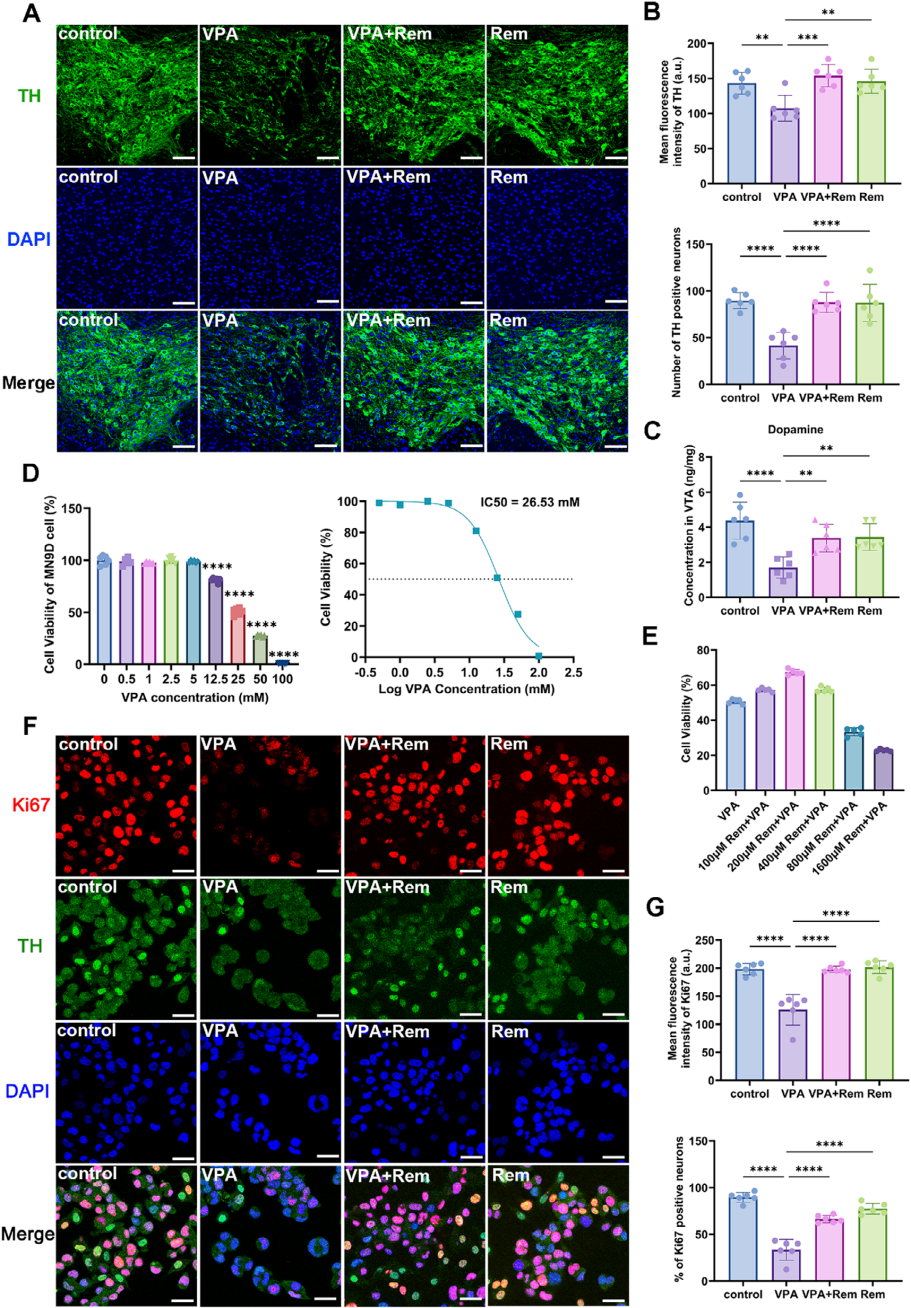
Remimazolam ameliorates VPA‐induced VTA dopaminergic neuron damage. (A) Representative immunofluorescence images of TH (green) and DAPI (blue) in the VTA of VPA‐exposed mice. Scale bar = 50 µm (B) Mean fluorescence intensity of TH and the percentage of TH‐positive neurons in VTA. *n* = 6 per group. (C) Dopamine concentration in VTA measured by ELISA kits. *n* = 6 per group. (D) The cell viability of MN9D dopaminergic neuron detected after different concentration of VPA injection. *n* = 6 per group. (E) The cell viability of MN9D dopaminergic neuron detected after remimazolam and VPA adding to the cells. *n* = 6 per group. (F) Representative immunofluorescence images of Ki67 (red), TH (green) and DAPI (blue) in MN9D dopaminergic neuron. Scale bar = 25 µm. (G) Statistical graph of mean fluorescence intensity and positive neurons. *n* = 6 per group. Values was presented as mean ± SEM and analyzed by one‐way ANOVA or Two‐way ANOVA. ns > 0.05, ^*^ *p* < 0.05, ^**^ *p* < 0.01, ^***^ *p* < 0.001 and ^****^ *p* < 0.0001.

To determine if the therapeutic effects of remimazolam were GABA‐dependent, we pre‐treated mice with the specific GABA_A receptor antagonist flumazenil. Surprisingly, flumazenil failed to block the behavioral improvements, indicating that remimazolam's action in this model is not primarily mediated by canonical GABAergic signaling (Figure S1). Furthermore, no significant changes in the number of GABAergic neurons or in GAD67 expression within the VTA were found (Figure S2). In contrast, the density of tyrosine hydroxylase‐positive (TH+) dopaminergic neurons—the most abundant neuronal population in the VTA—was significantly reduced in VPA‐exposed mice. Remimazolam treatment prevented this loss of dopaminergic neurons. (Figure 4A,B). Furthermore, we discovered a decrease in dopamine protein expression in VTA tissues exposed to VPA, further demonstrating damage to the dopamine system, which were improved by remimazolam (Figure 4C). To further validate the protective effect of remimazolam on dopamine neurons, we performed experiments on MN9D dopamine neuron cells. Firstly, we found that the IC50 of VPA to MN9D was 26 mм by CCK8 assay (IC50 = 26.53 mм) (Figure 4D). Subsequently, we used 25 mм VPA as the default concentration for VPA‐induced MN9D dopamine neuron damage model. And it was verified that the optimal concentration of remimazolam for treating VPA‐induced dopamine neuron damage is 200 µм (Figure 4E). Remimazolam protected MN9D cells from the decrease of proliferation ability caused by VPA, further indicating that the protective effect of remimazolam on dopaminergic neurons (Figure 4F,G).

### 2.3 Chemogenetic Inhibition of VTA Dopaminergic Neurons Abrogated the Ameliorating Effects of Remimazolam on Autistic‐Like Behaviors

**FIGURE 5:**
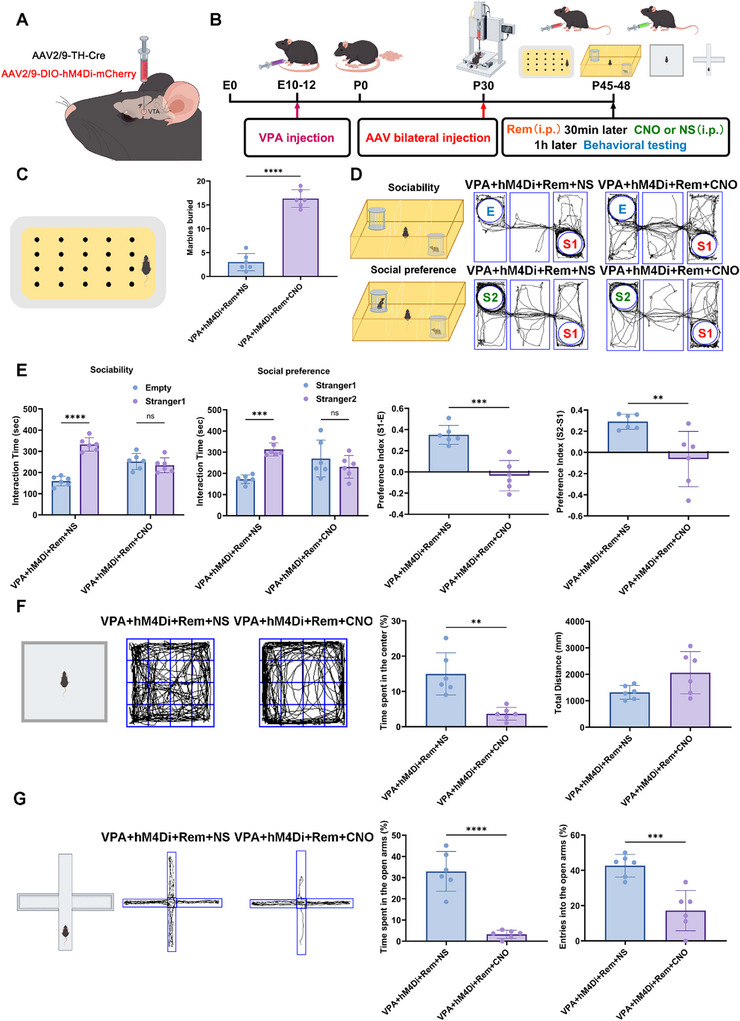
Chemogenetic inhibition of VTA dopaminergic neurons reversed the therapeutic effects of remimazolam on autistic‐like behaviors behaviors. (A) Schematic of Chemogenetic inhibition of VTA dopaminergic neurons in VPA‐exposed mice. (B) An outline of the experimental process for administering drugs and performing behavior measures. (C) The schematic diagram of marble burial experiment and the statistical graph of buried marbles number. *n* = 6 per group. (D) Representative traces of three‐chamber social interaction test (E: Empty, S1: stranger 1, S2: stranger 2). (E)Statistical graph of sociability and social novelty test. *n* = 6 per group (F) Representative traces of open‐field test. Statistical graph of the percent of time spent in the center and total distance. *n* = 6 per group. (G) Representative traces of elevated plus maze test. Statistical graph of time and entries spent in open arms. *n* = 6 per group. Values was presented as mean ± SEM and analyzed by one‐way ANOVA or Two‐way ANOVA. ns > 0.05, ^*^ *p* < 0.05, ^**^ *p* < 0.01, ^***^ *p* < 0.001 and ^****^ *p* < 0.0001.

To further validate the role of VTA dopaminergic neurons in remimazolam's ameliorative effects, chemogenetic approach via bilateral VTA infection with AAV2/9‐DIO‐hM4Di‐mCherry and AAV2/9‐TH‐Cre was used to inhibit VTA dopaminergic neurons (Figure 5A). Behavioral assessments were conducted after a 2‐week viral expression and subsequent administration of remimazolam and CNO. (Figure 5B). Targeted silencing of dopaminergic neurons after CNO injection eliminated remimazolam's ameliorative effects on stereotyped behaviors and social impairment in VPA‐exposed mice (Figure 5C–E). Furthermore, targeted silencing of dopaminergic neurons in CNO‐treated mice induced hyperactivie, stereotyped (circling), and anxious behaviors in the open field test, further suggesting the critical role of VTA dopaminergic neurons in the remimazolam treatment of autistic‐like behaviors (Figure 5F). Consistently, the CNO‐treated VPA‐exposed mice exhibited significant anxiety‐like behavior, characterized by a pronounced avoidance of the open arms. (Figure 5G). The above results suggest that VTA dopamine neurons play a critical role in remimazolam's ameliorative effects on ASD.

**FIGURE 6:**
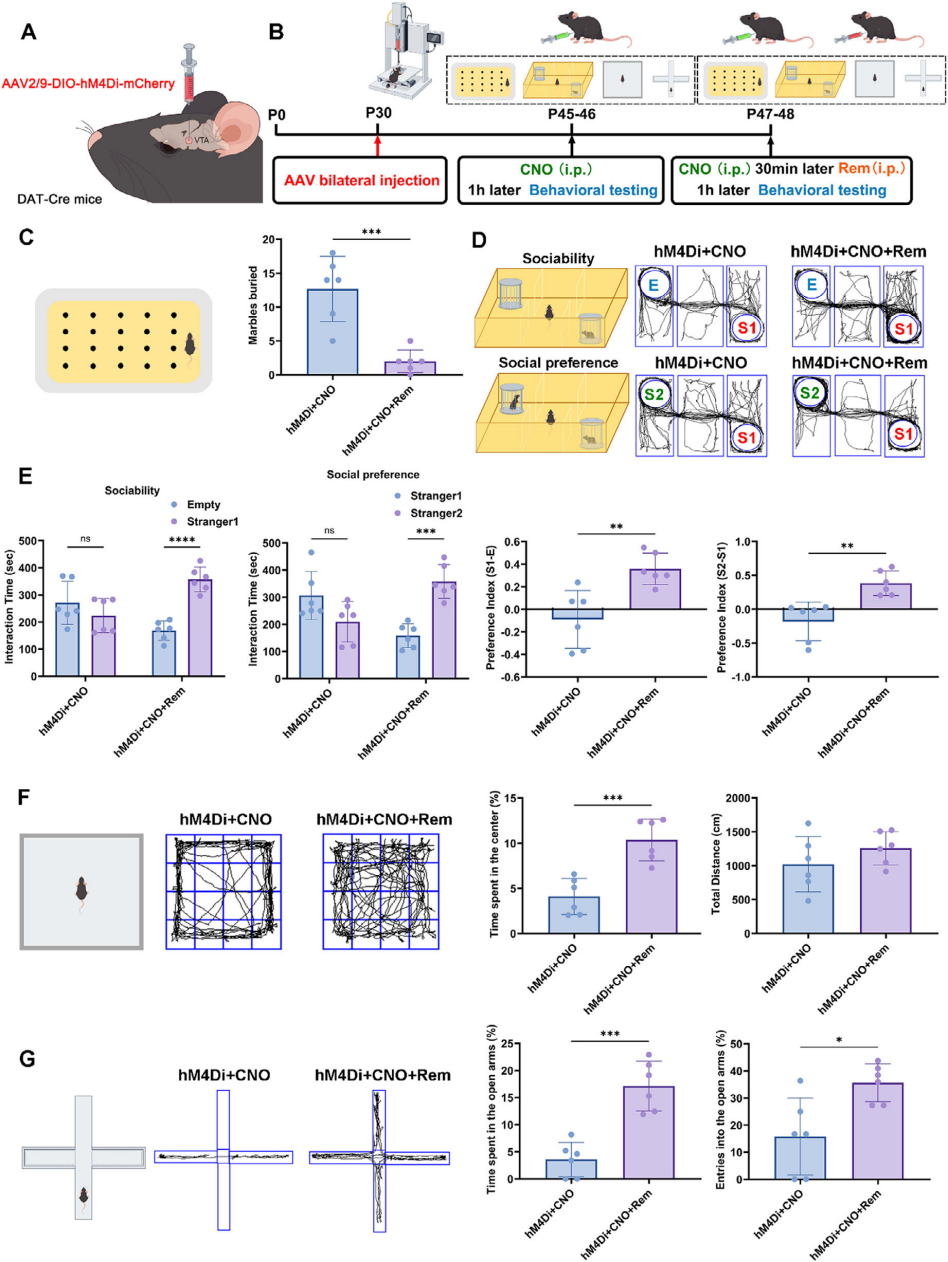
Chemogenetic suppression of VTA dopaminergic neurons in DAT Cre mice generated autistic‐like symptoms that were ameliorated by remimazolam. (A) Schematic of Chemogenetic inhibition of VTA dopaminergic neurons in DAT Cre mice. (B) An outline of the experimental process for administering virus and drugs and performing behavior measures. (C) The schematic diagram of marble burial experiment and the statistical graph of buried marbles number. *n* = 6 per group. (D) Representative traces of three‐chamber social interaction test (E: Empty, S1: stranger 1, S2: stranger 2). (E)Statistical graph of sociability and social novelty test. *n* = 6 per group (F) Representative traces of open‐field test. Statistical graph of the percent of time spent in the center and total distance. *n* = 6 per group. (G) Representative traces of elevated plus maze test. Statistical graph of time and entries spent in open arms. *n* = 6 per group. Values was presented as mean ± SEM and analyzed by one‐way ANOVA or Two‐way ANOVA. ns > 0.05, ^*^ *p* < 0.05, ^**^ *p* < 0.01, ^***^ *p* < 0.001 and ^****^ *p* < 0.0001.

Furthermore, we bilaterally injected the VTA of DAT‐Cre mice with AAV2/9‐DIO‐hM4Di‐mCherry (Figure 6A). After 2 weeks of recovery, the mice were treated with CNO intraperitoneal injection, and the behavioral observation was performed 1 h later (Figure 6B). In the marble‐burying experiment, targeted inhibition of dopaminergic neurons in the VTA after CNO treatment led to a marked increase in stereotypic behavior in mice, while remimazolam treatment attenuated CNO‐induced stereotypic behavior (Figure 6C). Furthermore, targeted chemogenetic suppression of VTA dopaminergic neurons in DAT Cre mice resulted in deficiencies in sociability and social preference, which were restored by remimazolam therapy (Figure 6D,E). In the open field test, chemogenetic inhibition of VTA dopaminergic neurons led to marked anxiety in DAT Cre mice, specifically as a fear to enter the central region of the open field (Figure 6F). Similarly, targeted inhibition of VTA dopaminergic neurons made DAT Cre mice anxious and afraid to explore the open arm region during the elevated plus maze test (Figure 6G). The above results further indicate that the functional decline of dopaminergic neurons in VTA is an important cause of autistic‐like behaviors, and remimazolam exerts its therapeutic effect on autistic‐like behaviors by improving the function of dopaminergic neurons in VTA.

### 2.4 Remimazolam Alleviates ASD via Suppression of Ferroptosis of Dopamine Neurons

To further explore the specific mechanism by which remimazolam protects the number and function of VTA dopaminergic neurons to improve autistic‐like behaviors in VPA‐exposed mice, we first conducted apoptosis detection experiments. Annexin V flow cytometry results showed that the apoptosis of dopamine neurons induced by VPA could be alleviated by remimazolam (Figure S3A). Subsequently, we performed apoptosis pathway‐related protein detection, and found that BAX, Bcl‐2 and Caspase 3 in the classic apoptosis pathway did not change significantly, and the results were not statistically different, suggesting that there are other potential pathways (Figure S3B).

**FIGURE 7:**
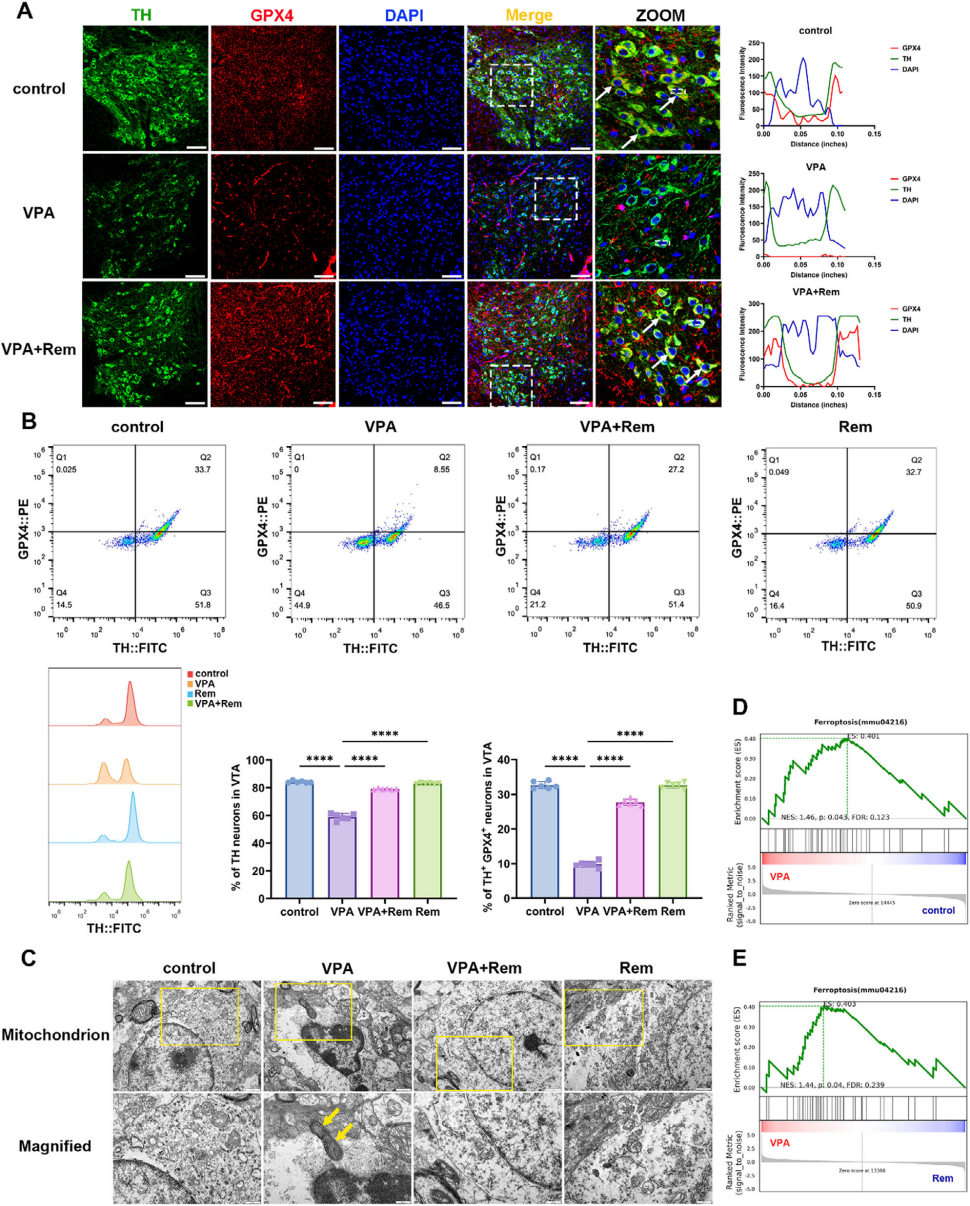
Remimazolam ameliorated the decrease of ferroptosis marker GPX4 and mitochondrial damage in VTA dopaminergic neurons of VPA‐exposed mice. (A) Representative immunofluorescence images of TH (green) and GPX4 (red) in VTA dopaminergic neuron in VPA‐exposed mice. Scale bar = 50 µm. Fluorescence co‐localization analysis diagram of TH and GPX4. (B) Representative plots of TH (FITC) and GPX4 (PE) cells present in the VTA brain region of mice were examined by flow cytometry. Peak plots of TH cells and GPX4 cells. Statistical plots of the proportion of TH cells and the percentage of TH^+^GPX4^+^ cells. *n* = 6 per group. (C) Mitochondrial morphology of dopaminergic neuron in VTA viewed under electron microscope. Scale bar = 500 nm. The yellow arrows indicate damaged mitochondria. (D‐E) GSEA enrichment analysis of ferroptosis pathway in different groups of RNA‐seq analysis. *n* = 3 per group. Values was presented as mean ± SEM and analyzed by one‐way ANOVA. ns > 0.05, ^*^ *p* < 0.05, ^**^ *p* < 0.01, ^***^ *p* < 0.001 and ^****^ *p* < 0.0001.

We next explored whether ferroptosis VTA dopaminergic neurons was involved in remimazolam's ameliorative effects on ASD. We first conducted tests at the animal level and found that the expression of GPX4, a core ferroptosis marker and regulator, was significantly reduced in the VTA dopamine neurons of VPA‐exposed mice was significantly reduced. While remimazolam significantly enhanced the expression of GPX4 in the VTA dopamine neurons of VPA‐exposed mice (Figure 7A). Futhermore, VPA‐exposed mice showed a substantial reduction in VTA dopamine neurons, especially the TH^+^GPX4^+^ subpopulation. Remimazolam effectively attenuated this neuronal loss, especially the TH^+^GPX4^+^ subpopulation in VPA‐exposed mice (Figure 7B). Mitochondrial shrinkage and cristae loss in the VTA dopamine neurons of VPA‐exposed mice were observed under the electron microscope andameliorated by remimazolam treatment (Figure 7C). Furthermore, RNA‐seq of VTA revealed significant alterations in the ferroptosis pathway following remimazolam administration (Figure 7D,E). Collectively, these results strongly suggested that the ferroptosis pathway was activated in VTA dopamine neurons of VPA‐exposed mice and remimazolam confered protection by suppressing this pathway, thereby restoring dopamine neuron number and function.

**FIGURE 8:**
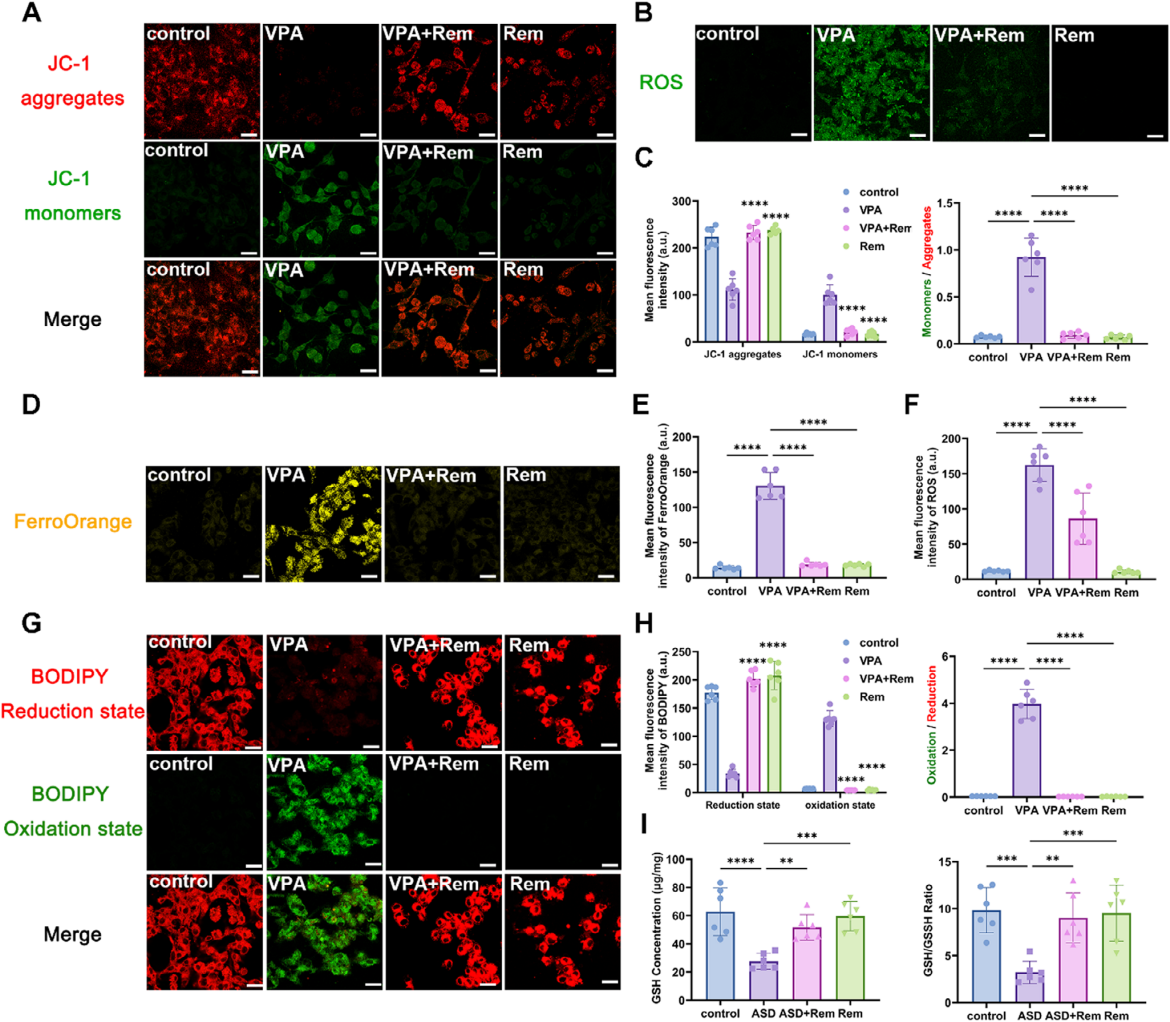
Remimazolam ameliorated the distinctive alterations of dopamine neuron ferroptosis caused by VPA. (A) Representative immunofluorescence images of JC‐1 monomers (green) and JC‐1 aggregates (red) in MN9D dopaminergic neuron. Scale bar = 25 µm. (B) Representative immunofluorescence images of ROS (green) in MN9D dopaminergic neuron. Scale bar: 25 µm. (C) Mean fluorescence intensity of JC‐1 and ratio of monomers and aggregates. *n* = 6 per group. (D) Representative immunofluorescence images of FerroOrange (orange) in MN9D dopaminergic neuron. Scale bar = 25 µm. (E) Mean fluorescence intensity of FerroOrange. *n* = 6 per group. (F) Mean fluorescence intensity of ROS. *n* = 6 per group. (G) Representative immunofluorescence images of BODIPY Oxidation state (green) and BODIPY Reduction state (red) in MN9D dopaminergic neuron. Scale bar: 25 µm. (H) Mean fluorescence intensity of BODIPY and ratio of Oxidation state and Reduction state. *n* = 6 per group. (I) GSH concentration and the ratio of GSH and GSSH in VTA measured by ELISA kits. *n* = 6 per group. Values was presented as mean ± SEM and analyzed by one‐way ANOVA or Two‐way ANOVA. ns > 0.05, ^*^ *p* < 0.05, ^**^ *p* < 0.01, ^***^ *p* < 0.001 and ^****^ *p* < 0.0001.

**FIGURE 9:**
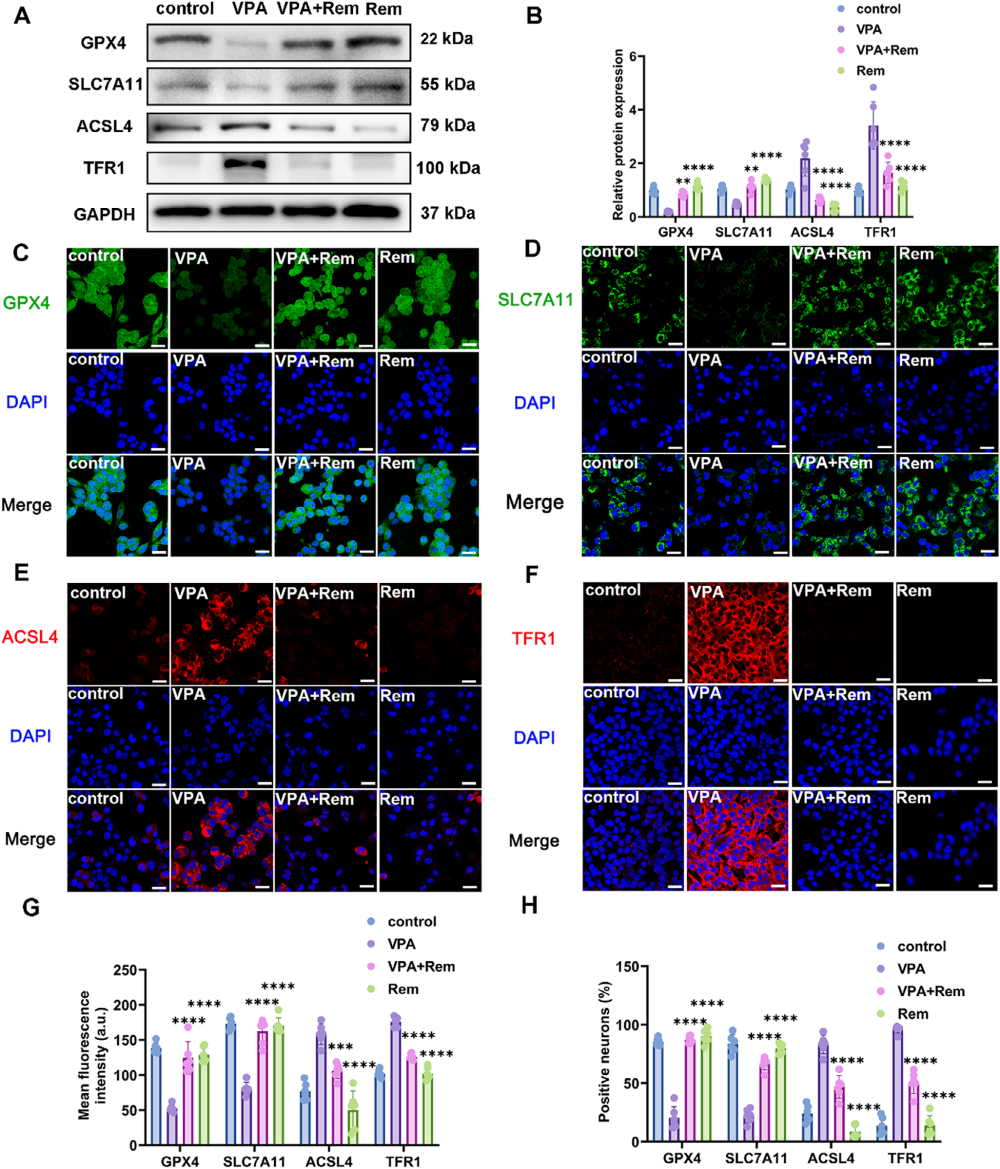
Remimazolam improved the key proteins in VPA‐induced ferroptosis of dopamine neurons. (A) Representative protein bands of GPX4, SLC7A11, ACSL4 and TFR1 in MN9D dopaminergic neuron. (B) Statistical graph of protein levels of GPX4, SLC7A11, ACSL4 and TFR1. *n* = 6 per group. (C) Representative immunofluorescence images of GPX4 (green) and DAPI (blue) in MN9D dopaminergic neuron. Scale bar = 25 µm. (D) Representative immunofluorescence images of SLC7A11 (green) and DAPI (blue) in MN9D dopaminergic neuron. Scale bar = 25 µm. (E) Representative immunofluorescence images of ACSL4 (red) and DAPI (blue) in MN9D dopaminergic neuron. Scale bar = 25 µm. (F) Representative immunofluorescence images of TFR1 (red) and DAPI (blue) in MN9D dopaminergic neuron. Scale bar = 25 µm. (G) Mean fluorescence intensity of GPX4, SLC7A11, ACSL4 and TFR1. *n* = 6 per group. (H) Statistical graph of positive neurons of GPX4, SLC7A11, ACSL4 and TFR1. *n* = 6 per group. Values was presented as mean ± SEM and analyzed by one‐way ANOVA or Two‐way ANOVA. ns > 0.05, ^*^ *p* < 0.05, ^**^ *p* < 0.01, ^***^ *p* < 0.001 and ^****^ *p* < 0.0001.

Prompted by the evidence of ferroptosis from our in vivo studies, we further investigated whether remimazolam protect MN9D cells from VPA‐induced ferroptosis. One of the most important alterations that occurs during ferroptosis is mitochondrial modification. We detected the decrease of mitochondrial membrane potential of dopamine neurons induced by VPA, and the change of mitochondrial membrane potential could be restored by remimazolam (Figure 8A,C). ROS promotes lipid peroxidation and plays a key role in ferroptosis, and we found that VPA caused a significant increase in ROS in dopamine neurons, while remimazolam down‐regulated the increased ROS and reduced peroxidation (Figure 8B,F). Iron accumulation is one of the important signs of ferroptosis, which is reflected by the detection of free ferrous ions. The results showed that remimazolam reduced the increase of ferrous ions in dopamine neurons induced by VPA and eliminated iron overload (Figure 8D,E). According to the BODIPY test results, remimazolam reduced lipid peroxidation induced by VPA and protected dopamine neurons (Figure 8G,H). Furthermore, it was shown that remimazolam corrected the negative changes caused by VPA, which included a drop in reduced glutathione and a decrease in the ratio of reduction to oxidation (Figure 8I). Subsequently, we also detected the protein levels of several key proteins that play an important role in ferroptosis: GPX4, SLC7A11, ACSL4 and TFR1. We found that both WB and IF, remimazolam effectively improved the protein level changes caused by VPA (Figure 9A–H). These results indicated that remimazolam alleviated autism spectrum disorder via suppression of ferroptosis of VPA‐induced dopamine neurons.

### 2.5 Ferroptosis Agonist Reversed the Treatment of Remimazolam on Ferroptosis in VPA‐Exposed Dopaminergic Neurons

**FIGURE 10:**
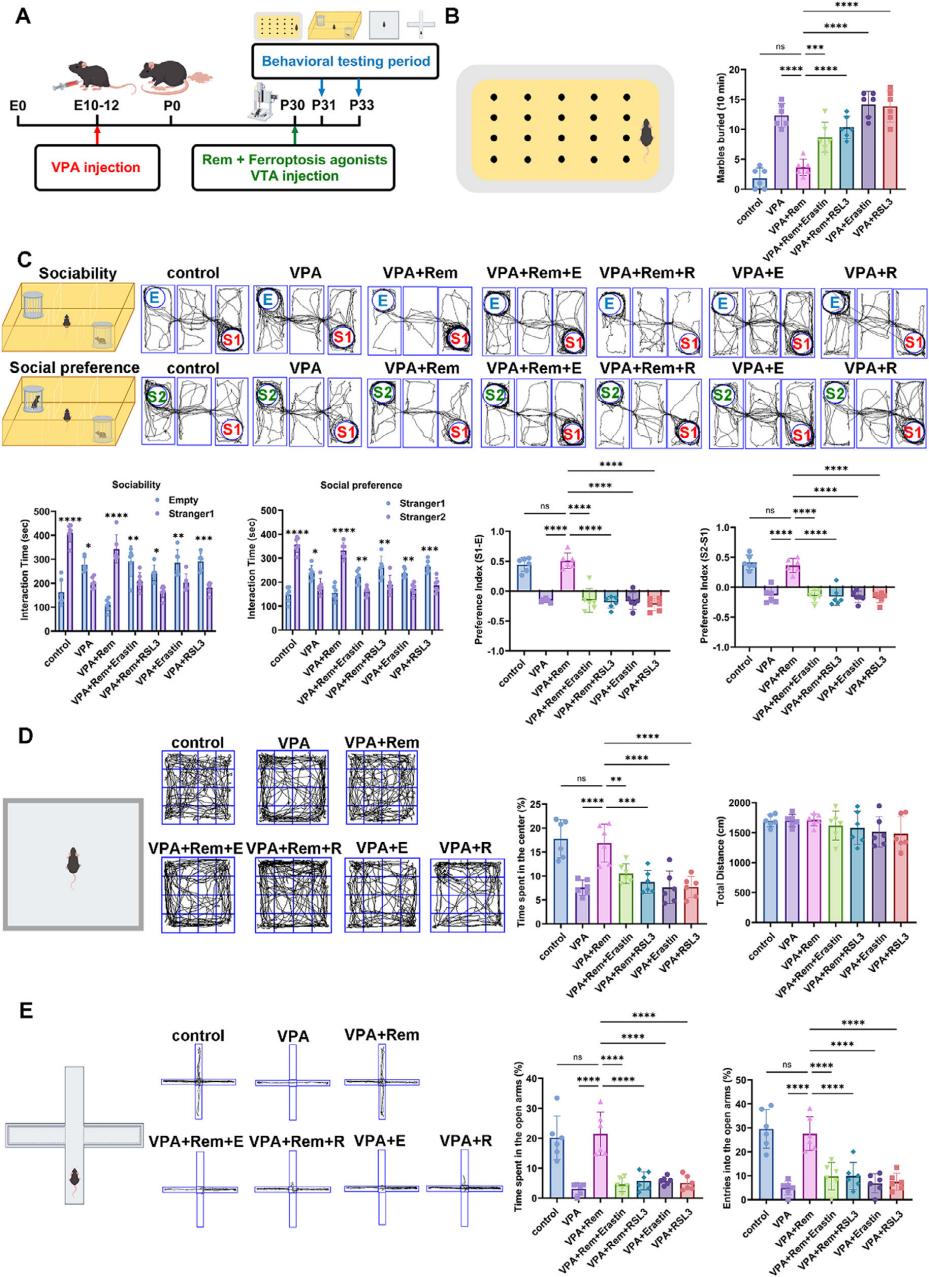
Ferroptosis agonist reversed the therapeutic effect of remimazolam on autism‐related behaviors in VPA‐exposed mice. (A) An outline of the experimental process for administering drugs and performing behavior measures. (B) The schematic of marble burial experiment and the statistical graph of buried marbles number. *n* = 6 per group. (C) Representative traces of three‐chamber social interaction test (E: Empty, S1: stranger 1, S2: stranger 2). Statistical graph of sociability and social novelty test. *n* = 6 per group (D) Representative traces of open‐field test. Statistical graph of the percent of time spent in the center and total distance. *n* = 6 per group. (E) Representative traces of elevated plus maze test. Statistical graph of time and entries spent in open arms. *n* = 6 per group. Values was presented as mean ± SEM and analyzed by one‐way ANOVA or Two‐way ANOVA. ns > 0.05, ^*^ *p* < 0.05, ^**^ *p* < 0.01, ^***^ *p* < 0.001 and ^****^ *p* < 0.0001.

**FIGURE 11:**
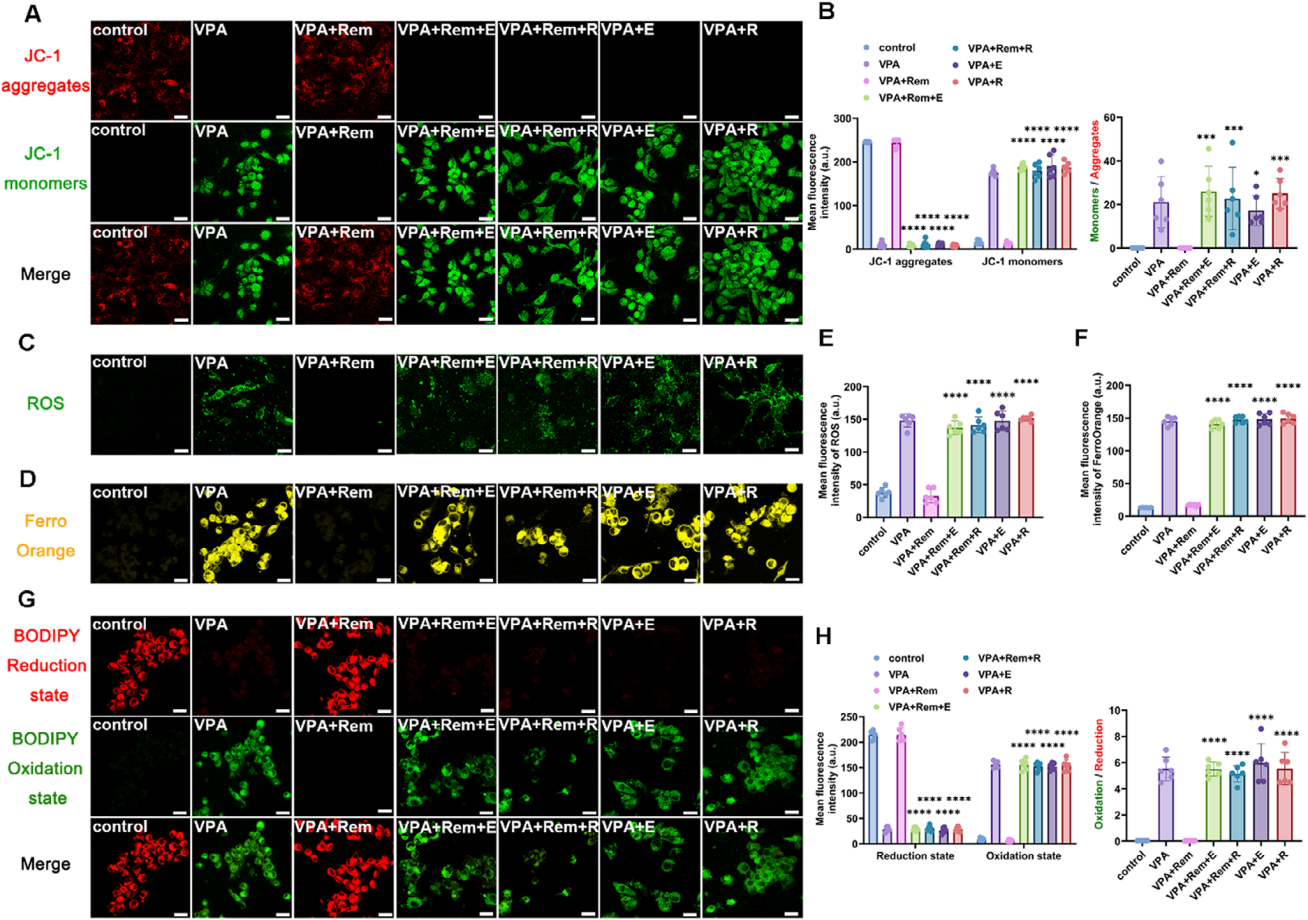
Ferroptosis agonist reversed the improvement of remimazolam on the characteristic changes of ferroptosis in VPA‐exposed dopaminergic neurons. (A) Representative immunofluorescence images of JC‐1 monomers (green) and JC‐1 aggregates (red) in MN9D dopaminergic neuron. Scale bar = 25 µm. (B) Mean fluorescence intensity of JC‐1 and ratio of monomers and aggregates. *n* = 6 per group. (C) Representative immunofluorescence images of ROS (green) in MN9D dopaminergic neuron. Scale bar = 25 µm. (D) Representative immunofluorescence images of FerroOrange (orange) in MN9D dopaminergic neuron. Scale bar = 25 µm. (E) Mean fluorescence intensity of ROS. *n* = 6 per group. (F) Mean fluorescence intensity of FerroOrange. *n* = 6 per group. (G) Representative immunofluorescence images of BODIPY Oxidation state (green) and BODIPY Reduction state (red) in MN9D dopaminergic neuron. Scale bar = 25 µm. (H) Mean fluorescence intensity of BODIPY and ratio of Oxidation state and Reduction state. *n* = 6 per group. Values was presented as mean ± SEM and analyzed by one‐way ANOVA or Two‐way ANOVA. ns > 0.05, ^*^ *p* < 0.05, ^**^ *p* < 0.01, ^***^ *p* < 0.001 and ^****^ *p* < 0.0001.

**FIGURE 12:**
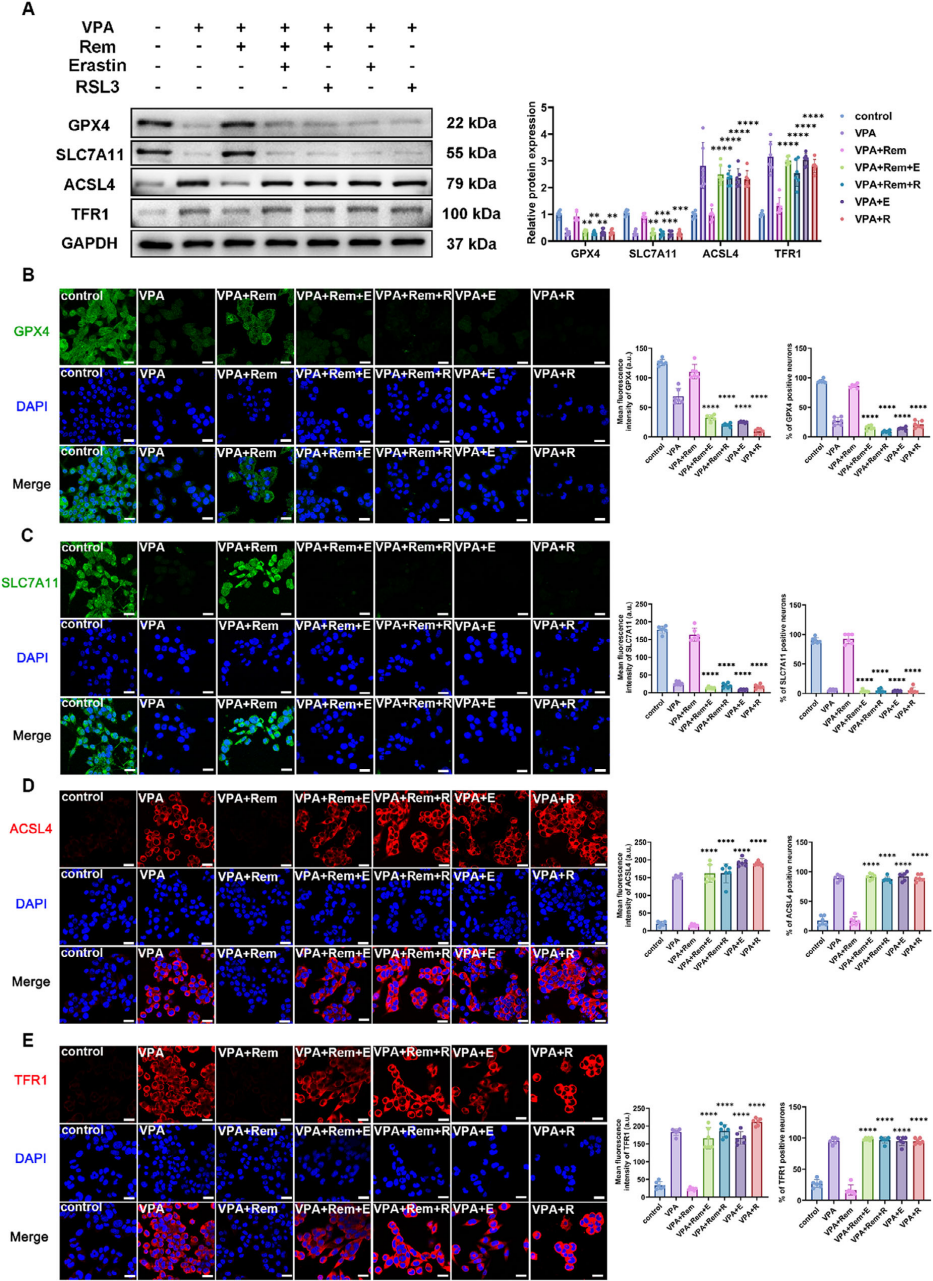
Ferroptosis agonist reversed the improvement of remimazolam on the key proteins of ferroptosis pathway in VPA‐exposed dopaminergic neurons. (A) Representative protein bands of GPX4, SLC7A11, ACSL4 and TFR1 in MN9D dopaminergic neuron. Statistical graph of protein levels of GPX4, SLC7A11, ACSL4 and TFR1. *n* = 6 per group. (B) Representative immunofluorescence images of GPX4 (green) and DAPI (blue) in MN9D dopaminergic neuron. Statistical graph of mean fluorescence intensity and positive neurons of GPX4. *n* = 6 per group. Scale bar = 25 µm. (C) Representative immunofluorescence images of SLC7A11 (green) and DAPI (blue) in MN9D dopaminergic neuron. Statistical graph of mean fluorescence intensity and positive neurons of SLC7A11. *n* = 6 per group. Scale bar = 25 µm. (D) Representative immunofluorescence images of ACSL4 (red) and DAPI (blue) in MN9D dopaminergic neuron. Statistical graph of mean fluorescence intensity and positive neurons of ACSL4. *n* = 6 per group. Scale bar = 25 µm. (E) Representative immunofluorescence images of TFR1 (red) and DAPI (blue) in MN9D dopaminergic neuron. Statistical graph of mean fluorescence intensity and positive neurons of TFR1. *n* = 6 per group. Scale bar = 25 µm. Values was presented as mean ± SEM and analyzed by one‐way ANOVA or Two‐way ANOVA. ns > 0.05, ^*^ *p* < 0.05, ^**^ *p* < 0.01, ^***^ *p* < 0.001 and ^****^ *p* < 0.0001.

To further confirm remimazolam's role in the treatment of ASD by reversing ferroptosis in VTA dopamine neurons, we used two ferroptosis agonists: Erastin and RSL3 (Figure 10A). The protective effect of remimazolam on marble burial was successfully counteracted by both of the ferroptosis agonists (Figure 10B). Similarly, the therapeutic effects of social novelty and social preference were reversed by ferroptosis agonists (Figure 10C). The reversal effect of ferroptosis agonist on remimazolam treatment was also observed in the open field and elevated plus maze tests (Figure 10D,E). The above results provide evidence that the therapeutic effects of remimazolam on ASD do work by improving ferroptosis in VTA dopaminergic neurons. Ferroptosis agonists reversed the protective effect of remimazolam on mitochondrial membrane potential and aggravated lipid peroxidation and iron overload, which proved that the therapeutic effect of remimazolam was closely related to the reversal of ferroptosis in dopamine neurons (Figure 11A–H). Similarly, ferroptosis agonists effectively reversed the protective effect of remimazolam on key proteins in the ferroptosis pathway, demonstrating that VPA‐induced ferroptosis of VTA dopamine neurons is one of the important causes of ASD, and remimazolam plays a therapeutic role in ASD by protecting VTA dopamine neurons (Figure 12A–E).

Since ferroptosis in VTA dopaminergic neurons is one of the underlying mechanisms of ASD, we used two ferroptosis inhibitors to observe their therapeutic effects on ASD and compared their efficacy with remimazolam treatment (Figure S4A). Ferroptosis inhibitors alleviated the stereotypy induced by VPA exposure in the marble burial experiment, but the therapeutic effect was slightly less than that of remimazolam (Figure S4B). Meanwhile, ferroptosis inhibitors improve social impairment in ASD, but the overall effect is less than that of remimazolam (Figure S4C). Besides, ferroptosis inhibitor alone did not effectively alleviate the anxiety of VPA‐exposed mice in the open field test (Figure S4D). Finally, ferroptosis inhibitor fully alleviated the anxiety of VPA‐exposed mice in elevated plus maze (Figure S4E). These results indicate that both ferroptosis inhibitor and remimazolam are potential targeted drugs for the treatment of autism, but ferroptosis inhibitor is slightly less effective than remimazolam. In terms of ameliorating the characteristic changes of ferroptosis, both ferroptosis inhibitors and remimazolam effectively ameliorated VPA‐induced decrease in mitochondrial membrane potential, lipid peroxidation and increase in free iron ions (Figure S5A–H). In the key molecular proteins of ferroptosis pathway, ferroptosis inhibitor and remimazolam could effectively alleviate the adverse changes caused by VPA exposure (Figure S6A–E). These results suggest that inhibition of ferroptosis in VTA dopamine neurons may be one of the important mechanisms of remimazolam in the treatment of ASD, and there are other potential mechanisms involved in the therapeutic effect of remimazolam. Therefore, the behavioral therapeutic effect of remimazolam is generally better than that of ferroptosis inhibitors alone.

## 3 Discussion

**FIGURE 13:**
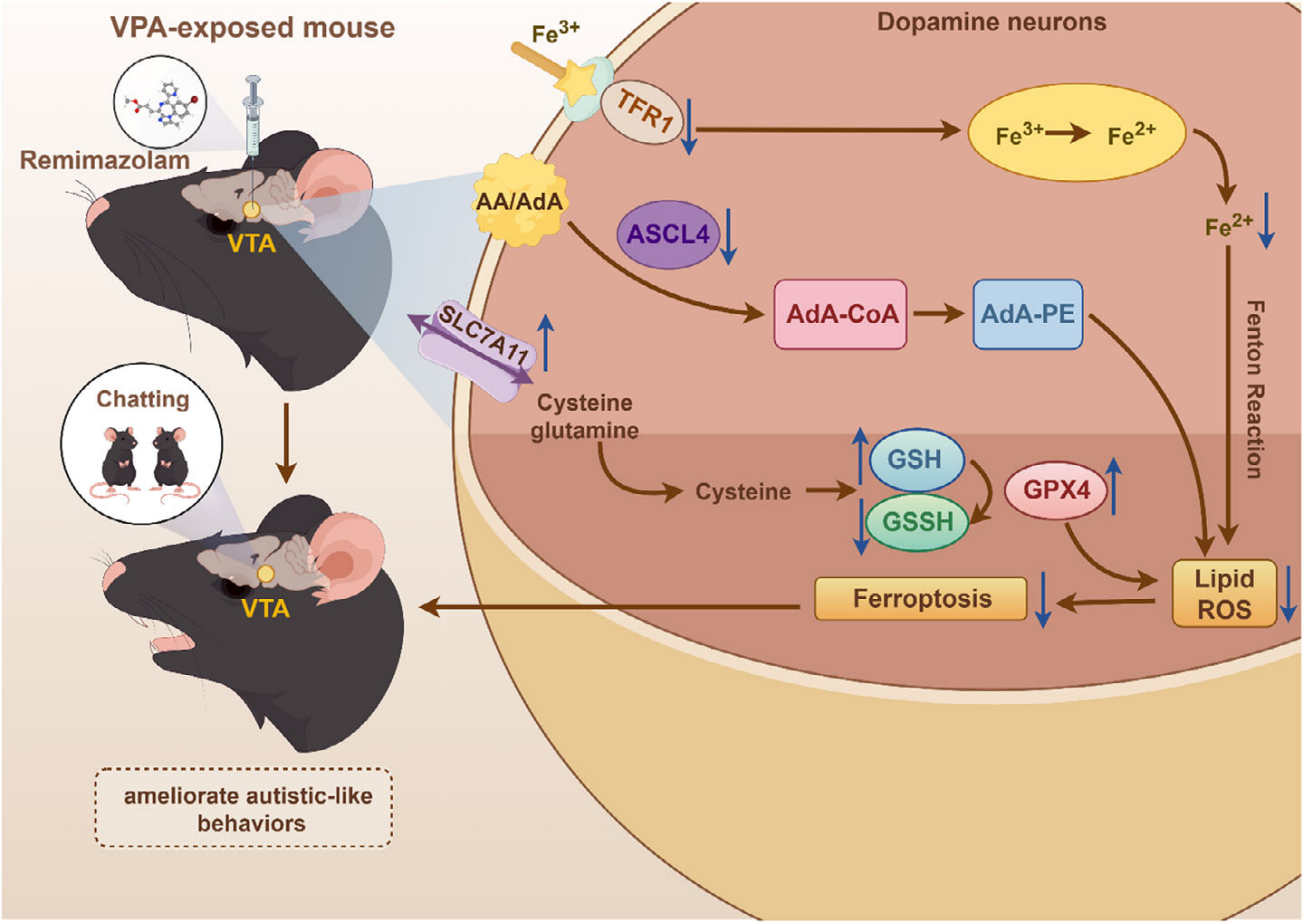
A schematic illustrating how remimazolam alleviates autistic‐like behaviors by suppressing ferroptosis in VTA dopaminergic neurons of VPA‐exposed mice. Figure created in Figdraw (www.figdraw.com).

This study demonstrated dose and duration effect of remimazolam in the treatment of autistic‐like behaviors in ASD, and unexpectedly found that the ultra‐short‐acting GABA agonist remimazolam was able to treat autistic‐like behaviors for up to 3–7 days. Meanwhile, we also found that remimazolam had a significant protective effect on dopamine neurons in the VTA of VPA‐exposed mice. This research mainly discovered that remimazolam ameliorated ASD by suppressing ferroptosis in VTA dopaminergic neurons (Figure 13).

In investigating the dose and duration of remimazolam in the treatment of autistic‐like manifestations, we found that higher doses of remimazolam within the safety range exert better therapeutic effects, and unexpectedly found that a single intraperitoneal injection of remimazolam could provide a lasting 3–7 days therapeutic effects. Remimazolam has been proven to have other long‐term therapeutic effects besides sedation, such as protecting cognitive function and treating bone cancer pain, but no previous studies have shown the effect of remimazolam on ASD [41, 42]. As an ultra‐short‐acting drug, remimazolam's therapeutic effects on ASD lasting 3–7 days and remaining unaffected by flumazenil may indicate potential mechanisms independent of its primary target, the GABA receptor.

Moreover, we found significant reduction of TH^+^ dopaminergic neurons in the VTA of VPA‐exposed mice by immunofluorescence and flow cytometry, which may be mitigated following remimazolam treatment. The experimental results were consistent with the results of some previous studies, but other studies showed no statistical difference. [17, 18] The possible reasons for the inconsistent results may include different methods for establishing ASD animal models, different animal species such as rats and mice, and different detection times and methods. Although a reduction in TH^+^ cell number typically indicates the loss of dopaminergic neurons. However, it may also result from reversible inhibition of TH expression or delayed neuronal maturation. Furthermore, growing evidence suggests that dopaminergic neuron degeneration in VPA‐exposed mice is more likely a progressive process that begins immediately after VPA exposure and gradually emerge across postnatal stages. [17, 43] Therefore, we think remimazolam may temporarily halt ongoing dopaminergic neuron degeneration, rescue impaired but not yet fully degenerated neurons, and restore their function, leading to normalized TH expression reflected by an increase in TH^+^ cells. Studies have shown that Annexin V apoptosis detection positive did not only exist in the apoptotic pathway, ferroptosis also make Annexin V apoptosis appear false positive, which explained the contradiction of the above experiments. [44] This point is sufficient to explain the contradictory results of our Annexin V results showing apoptosis but WB results showing no apoptosis. In the cell experiments of this study, it was found that remimazolam could directly protect dopamine neurons to alleviate the damage caused by VPA exposure, which means that remimazolam may directly act on dopamine neuron receptors or temporarily improve abnormal key proteins.

The mechanism of remimazolam may also involve the enhanced function of VTA dopaminergic neurons in VPA‐exposed mice. Our chemogenetic experiments demonstrated a central role for VTA dopaminergic neurons in mediating remimazolam's amelioration of autistic‐like behaviors. Chemogenetic inhibition of VTA dopaminergic neurons abolishes remimazolam's amelioration of autistic‐like behaviors in VPA‐exposed mice. Furthermore, chemogenetic inhibition of VTA dopaminergic neurons can induce autistic‐like behaviors in DAT‐Cre mice, and remimazolam treatment reverses these behaviors. Behavioral improvements from remimazolam in autistic‐like behaviors likely result from a combination of compensatory hyperfunction in existing neurons, functional recovery of impaired neurons, and potential neural circuit compensation.

Accumulated evidence suggests that ferroptosis may be one of the underlying mechanisms of ASD [45, 46]. Our immunofluorescence and flow cytometry results in the VTA of VPA‐exposed mice showed a decrease in dopaminergic neurons, which was directly related to ferroptosis and could be mitigated by remimazolam. This fully indicates that ferroptosis of dopamine neurons exists in the VTA of VPA‐exposed mice and remimazolam plays a therapeutic role by improving ferroptosis of dopamine neurons. Ferroptosis agonist reversed the therapeutic effects of remimazolam on autistic‐like behaviors in VPA‐exposed mice and ferroptosis in MN9D dopamine cells, further supporting the role of remimazolam in ameliorating ASD by reducing ferroptosis of dopamine neurons. Studies have shown that VPA directly drives neuronal ferroptosis by upregulating DDIT4 expression and inhibiting the PI3K/Akt pathway and ferroptosis occurs in the hippocampus and cerebellum of VPA‐exposed mice at P12‐15 [38, 40, 45]. Combined with our findings of ferroptosis in VTA at P30, we speculate that ferroptosis is more likely an ongoing process that triggered by VPA exposure and emerge progressively across postnatal stages. Remimazolam may temporarily halt ongoing ferroptosis, rescue impaired but not yet fully dead neurons, and restore their function, leading to phenotypic recovery and behavioral improvements. However, after drug withdrawal, pre‐existing pathological conditions and fragile compensatory defenses may lead to the recurrence of dopaminergic neuron ferroptosis, resulting in the reappearance of autistic‐like behaviors. This study also further explored the therapeutic effect of ferroptosis inhibitors on ASD. Although there was significant relief, the results were not as good as that of remimazolam, which reflects that in addition to the reduction of ferroptosis, there are other therapeutic mechanisms of remimazolam that need to be further explored.

This study still has some limitations: Firstly, whether remimazolam exerts its neuroprotective and therapeutic effects on autistic‐like behaviors by directly or indirectly acting on dopaminergic neurons and the specific molecular mechanisms are still unclear, which is the focus of our upcoming study. Second, due to the difficulty in breeding and obtaining offspring ASD models in this study, the male and female group control study was not conducted. Some studies have shown that gender is an important factor affecting autism‐like behavior and the number of dopamine neurons [47]. The groups in this study were selected in a 1:1 ratio of male and female to minimize the significant differences caused by gender.

## 4 Conclusion

The experimental results showed that remimazolam significantly alleviated the core symptoms of ASD with a lasting effect. VTA dopaminergic neurons may be the key neurons of remimazolam in the treatment of ASD. Remimazolam ameliorates autistic manifestations via suppression of ferroptosis in VTA dopaminergic neurons.

## 5 Experimental Section

### 5.1 Animals

The source of the clean C57BL/6L mice (6–8 weeks) was Shanghai Xinuogu Experimental Animal Co., Ltd. The DAT Cre mice (RRID: IMSR_JAX: 006660, 6–8 weeks) were purchased from Genepax Biotechnology Co., Ltd. In the clean animal department, cages typically held four to five animals. Free drinking and feeding are provided, along with a 12‐hour light/dark cycle, 24±2°C temperatures, and 50% ± 10% relative humidity. A week was spent acclimating the experimental animals to the conditions of the animal room. In terms of animal mating, we place a male and two female mice in a cage at night, and check the females' vaginal openings the following morning to see if a pregnancy plug is present. Animal diet and experimental procedures were strictly adhered to, in accordance with the Shanghai Sinogene's Experimental Animal Ethics Committee (permission number XNG201‐2310‐005) and international standards for experimental animal ethics and animal welfare.

### 5.2 VPA Administration and ASD Model

The experiment began after the newly purchased mice acclimated to the animal house for a week. The time of pregnancy was calculated when the female mice of 8 weeks saw the pregnant plug after mating. On the 10.5–12.5 days of pregnancy, the pregnant mice were intraperitoneally injected with valproate (VPA) (HY‐10585A, MCE) 600 mg kg^−1^. After fetal VPA‐exposed mice reached 30 days of age, corresponding drug injection, virus injection and behavioral detection were performed.

### 5.3 In Vivo Pharmacological Experiments

After VPA‐exposed neonatal mice grew up to 30 days, remimazolam was injected intraperitoneally or intracerebrally, and other drugs included Flumazenil, ferroptosis agonists and ferroptosis inhibitors. Remimazolam (Jiangsu Hengrui Medicine Co., Ltd. Jiangsu, China) was dissolved in sterile 0.9% NaCl solution and injected intraperitoneally (15 mg kg^−1^ and 30 mg kg^−1^) or intracerebrally (600 µм, 200 nL) to mice. The concentration of flumazenil injection (Zhejiang Autokang Pharmaceutical Group Co. LTD, Zhejiang, China) was 1 mg kg^−1^. Ferroptosis agonists: Erastin (10 µм, HY‐15763, MCE) and RSL3 (1 µм, HY‐100218A, MCE) or ferroptosis inhibitors: Ferrostatin‐1 (2 µм, HY‐100579, MCE) and Liproxstation‐1 (20 µм, HY‐12726, MCE) were dissolved in corn oil according to the instructions of MCE and previous researches.

### 5.4 In Vitro Pharmacological Experiments

MN9D dopamine neurons were inoculated in 6‐well plates (for WB) or 24‐well plates (for IF). After they grew for 24 h and reached an appropriate density, the corresponding drugs VPA (25 mм, HY‐10585A, MCE), Remimazolam (200 µм, Yichang Renfu Pharmaceutical Co. LTD), Erastin (10 µм, HY‐15763, MCE), RSL3 (1 µм, HY‐100218A, MCE), Ferrostatin‐1 (2 µм, HY‐100579, MCE) and Liproxstation‐1 (20 µм, HY‐12726, MCE) were added according to the experimental requirements. Following a 12‐hour drug stimulation period, cells were collected for further procedures in accordance with WB or IF.

### 5.5 Marble Burying Test

Mice with restricted repetitive behavior were evaluated using the marble burying test [48, 49]. It is surrounded by a box of acrylic material with a bottom and no top, so as to prevent mice from escaping, and a soft cushion material is placed under the bottom of the box for mice to bury. Twenty glass beads were uniformly distributed throughout the cage. The mice were all carefully put in the same cage corner and given freedom to roam around. The mice were taken out after 20 min, and the percentage of marble balls buried in padding material (>67%) was tallied.

### 5.6 Three‐Chamber Social Interaction Test

To evaluate social deficiencies in mice, a three‐chamber test was employed [49]. An opaque box with two transparent panels dividing it into three equal‐sized compartment was used for the test. There were three phases to the test. Mice in the first stage were given 10 min to investigate three compartments. A circular metal cage with a strange mouse (Stranger 1, S1) was positioned in a marginal region of a lateral compartment during the second sociability stage. In another lateral compartment, on the opposite side of S1, was an empty coop (Empty, E). Mice were permitted to freely explore the three compartments for 10 min. Another strange mouse (Stranger 2, S2) was used in place of the block during the social novelty period. Mice were given 10 min to freely explore the three compartments, same like in stage 2. The following equation is used to calculate the social preference index: social preference index = (TS1 ‐ TE) / (TS1 + TE) for sociability and (TS2 ‐ TS1) / (TS2 + TS1) for social novelty.

### 5.7 Open Field Test

The open field experiment was conducted in a glass box [50]. After spending 30–60 min in the room to acclimate, the experimental mice were carefully placed in the middle of the box and allowed to roam around for another half hour. The animal behavior monitor device automatically examined each mouse's behavioral path and total movement distance. The mouse's ability to exercise was assessed by measuring its overall movement distance, and its depression was assessed by measuring the amount of time it spent active in the central region.

### 5.8 Elevated Plus Maze

The elevated cross maze is made up of four extending arms that form a cross shape and a central platform [51]. In order to keep the animal from tumbling out of the maze, each arm typically features a boundary railing. Labyrinths often offer suitable lighting conditions and are situated in a comparatively calm setting. Animals must be pre‐trained to become accustomed to the maze's layout prior to the official experiment. This involves setting the animal up on a central platform so it may freely move throughout the maze. This phase aims to eradicate apprehension regarding the unfamiliar surroundings. The animals were positioned on the central platform for the official trial phase, after which they were free to roam around the maze's four arms. Software was used to examine the animals' trajectories after their activities in the maze were recorded for 5 min apiece. The number of times to enter the open arm and the duration of time spent there are the primary observation markers.

### 5.9 In Vivo Chemogenetic Manipulation

We purchased the necessary virus from BrainVTA: AAV2/9‐TH‐NLS‐Cre‐WPRE‐pA and AAV2/9‐Ef1α‐DIO‐hM4D(Gi)‐mCherry‐WPREs‐pA [16]. Before virus injection, the mice were anesthetized with 1.5% isoflurane and fixed on a brain stereotaxic device. Mice's eyes can be protected with Vaseline. Primarily, AAV2/9‐TH‐Cre was bilaterally injected into VTA (AP: −3.08 mm; ML: ±0.5 mm; DV: −4.55 mm) of VPA‐exposed mice with 300nL each site. Subsequently, a bilateral injection of AAV2/9‐Ef1α‐DIO‐hM4D(Gi)‐mCherry‐WPREs‐pA were administered into VTA. After 2 weeks of rest, the expression of viral infection peaked. Initially, we administered remimazolam intraperitoneally to mice. After 30 min, they were intraperitoneally injected with CNO or saline. Behavioral testing was carried out an hour later. DAT Cre mice were treated with bilateral VTA (AP: −3.08 mm; ML: ±0.5 mm; DV: −4.55 mm) injection of AAV2/9‐Ef1α‐DIO‐hM4D(Gi) ‐mCherry‐WPREs‐pA. Following a 2‐week period of rest, DAT Cre mice were given CNO intraperitoneally, and behavioral tests were conducted 1 h later. Two days later, the CNO injection was repeated and remimazolam was injected half an hour later. The DAT Cre mice were left to rest for 1 h for behavioral testing.

### 5.10 Enzyme‐Linked Immunosorbent Assay (ELISA)

Mouse Dopamine (EM30642, Wellbi Biological Technology), GSSH (ET0003M, Wellbi Biological Technology), and GSH (ET0004M, Wellbi Biological Technology) ELISA kits were used to assess the amounts of dopamine, GSSH, and GSH in the VTA samples that were obtained. The directions were followed in order to complete the specific phases. Following the procedure, a microplate reader was used to detect each hole's absorbance (OD value) at a wavelength of 450 nm. The protein content in the associated sample was then computed using the standard curve.

### 5.11 Transmission Electron Microscopy (TEM)

After the mice were anesthetized with 0.3% pentobarbital, their brains were quickly removed and the VTA brain tissue was carefully separated as quickly as possible to prevent the destruction of the organelle structure [52]. After separation, the brain tissue was immediately fixed in 2.5% glutaraldehyde electron microscope fixative solution. The samples were dehydrated using graded alcohol solutions before being implanted in epoxy resin. A Leica Ultracut microtome was used to cut ultrathin sections (100 nm), which were then gathered on copper grids and stained with uranyl acetate and lead citrate using a Leica EM Stainer. In order to evaluate structural and morphological alterations in the mitochondria, including swelling and cristae, the slices were lastly inspected using a JEM 1010 transmission electron microscopy (JEOL Ltd., Japan) at an accelerating voltage of 100 kV.

### 5.12 Western Blotting

In accordance with earlier studies, we anesthetized mice, removed the VTA region of their brains, and then preserved them in liquid nitrogen. A tissue grinder is used to make the homogenate after the protease and phosphatase inhibitors have been added to the RIPA lysate. The supernatant is centrifuged twice at 12 000 rpm for 20 min each at 4°C. The supernatant protein content and sample volume were calculated using the BCA protein concentration assay kit. Denatured after boiling, then chilled to −20 degrees. After 120 min of electrophoresis at 80 V, the samples were moved to a nitrocellulose membrane (0.22 µm) at a steady current of 200 mA. TBST (pH 7.5) containing 5% bovine serum albumin was used to block the membrane for 2 h at room temperature. Subsequently, primary antibodies diluted with 5 % bovine serum protein TBST, including mouse GPX4 antibody (67763‐1‐Ig, proteintech), rabbit SLC7A11 antibody (26864‐1‐AP, proteintech), rabbit ACSL4 antibody (R24265, Zenbio), rabbit TFR1 antibody (R381603, Zenbio), rabbit BAX antibody (60267‐1‐g, proteintech), mouse Bcl‐2 antibody (60178‐1‐Ig, proteintech), rabbit Caspase three antibody (9661s, CST), mouse GAPDH antibody (60004‐1‐Ig, proteintech). After an overnight incubation at 4°C, the incubation box was placed at room temperature for 30 min before the membrane was washed. The bands were incubated with horseradish peroxidase (HRP) labeled secondary antibody at room temperature for 2 h, and ECL chemiluminescence was developed after washing the membrane. Gray value data was obtained by quantitatively analyzing the stripes utilizing ImageJ software. The target protein's gray value was adjusted following a comparison between the reference protein's and the internal reference protein's gray values. The final data was statistically calculated using Graphpad software to see if there was a statistical difference.

### 5.13 Cell Culture

The MN9D cell (RRID: CVCL_M067, Quicell) line was cultured in α‐MEM medium (PM150410B, Pricella) with 10% fetal bovine serum and 1% penicillin‐streptomycin. The MN9D cell were seeded in 10 cm petri dish until it grew to 80% and then passaged in next generation. After the cell state stabilized, the cells were implanted into 6‐well, 24 well, and 96 well plates according to different experimental requirements.

### 5.14 Cell Viability Assay

The cells were injected into 96‐well culture plates at a density of 1×10^4^ cells per well, and they were then left to be exposed to different concentrations of VPA and Remimazolam for a full day. After a 24‐hour treatment, cells were added to 100 µL of cell media per well, the CCK‐8 reagent was added, and the cells were incubated for 2 h at 37°C. Utilizing a microplate reader, the absorbance at 450 nm was determined.

### 5.15 JC‐1 Assay

The JC‐1 kit (C2003S, Beyotime) was used to detect changes in mitochondrial membrane potential. MN9D dopamine neuron cells were seeded on 24‐well plate slides, and after they grew to the appropriate concentration, drugs such as VPA and remimazolam were added successively. Prepare the working solution for the JC‐1 reagent after the medicine has been in effect for a full day. After aspirating the culture media, add 250 µL of the entire culture medium. To ensure complete mixing, add 250 µL of the JC‐1 working solution. Incubate for 20 min at 37°C. Once removed, add 500 µL of medium and wash it twice with JC‐1 buffer solution. Subsequently, observe it rapidly under a confocal microscope.

### 5.16 Reactive Oxygen Species (ROS) Detection

The reactive oxygen species detection kit (S0033M, Beyotime) uses the fluorescent probe DCFH‐DA for reactive oxygen species detection. Prepare the DCFH‐DA working solution and add it to the cells to be tested with the culture medium removed. The solution should fully cover the cells. After incubation at 37°C for 20 min, it can be observed under a confocal microscope after washing three times with serum‐free medium. If there is no ROS, no green fluorescence can be observed. However, in the presence of ROS, a stronger green fluorescence will be observed.

### 5.17 FerroOrange Determination of Ferrous Ions

The FerroOrange probe (F374, Dojindo) was used to detect free divalent iron ions [53]. First, prepare a 1 mmol L^−1^ FerroOrange working solution. After treatment, the cells to be tested were directly added to the working solution and incubated in the incubator at 37°C for 30 min. Then, the cells could be directly observed under the microscope. If there was an obvious orange light under microscope, it indicated that there was an excessive content of free iron ions.

### 5.18 Lipid Droplets Green Fluorescence Assay Kit with BODIPY 493/503

BODIPY 493/503 Assay Kit (C2053M, Beyotime) was used to check for the presence of lipid peroxidation. After incubating the cells in the working solution for 20 min, they were washed twice with PBS and examined under a microscope. The reduced state emits red light, while the oxidized state emits green light. Calculating the red‐to‐green ratio can reveal the lipid peroxidation situation.

### 5.19 Flow Cytometry of Mouse VTA Brain Tissue

Mice were anesthetized and euthanized, and their brains were rapidly isolated and placed in PBS that had been precooled on ice. Surface blood was removed by gentle shaking and washing in PBS. Bilateral VTA brain regions were separated using microscopic tweezers and placed in new PBS. The VTA brain regions of three mice were collected as a group in 6‐well plates and 1 mL of 0.25% trypsin was added. The tissue was quickly chopped up to 0.5–1mm [3] in size with ophthalmic scissors. It was placed in a 37°C cell incubator for 20 min and gently shaken every 5 min to detach the cells on the surface. Trypsin digestion was terminated by adding 2 mL of DMEM complete medium containing 10% FBS to 6‐well plates. The blow was repeated until it was a single cell suspension, and the residual tissue block was filtered through a 200‐mesh cell sieve. The samples were centrifuged at 1000 rpm for 5 min at 4°C, the supernatant was discarded, resuspended in PBS, washed once, and centrifuged again. After cell counting, the concentration was adjusted to 10^7^ cells per milliliter. Cells were fixed with 4% paraformaldehyde for 10 min and washed three times with PBS. Then, they were permeabilized with 0.3% Triton for 20 min and washed three times with PBS. After blocking for 1 h, rabbit monoclonal TH antibody (ab137869, abcam) and mouse GPX4 antibody (67763‐1‐Ig, proteintech) were incubated directly at room temperature for 2 h and washed three times with PBS. Secondary antibodies CoraLite488‐conjugated Goat Anti‐Rabbit antibody (SA00013‐1, proteintech) and CoraLite594‐conjugated Goat Anti‐Mouse antibody (SA00013‐3, proteintech) were further incubated at room temperature in the dark for 2 h. After cleaning with PBS for three times, the machine was started for detection.

### 5.20 Flow Cytometry of MN9D Cells

MN9D dopamine cells were seeded into 6‐well plates, and VPA, remimazolam and other drugs were added successively when the cells proliferated to 60% density. After being collected, cells were cleaned using cold PBS. Cells were treated with Annexin V and propyl iodide (PI) using the apoptosis kit (LABLEAD Trading Co., Ltd.) to examine apoptosis.

### 5.21 Transcriptomics Analysis

RNA extraction and high‐throughput transcriptome analysis were performed on the VTA brain regions of mice. Mice for transcriptome sequencing in this study were divided into control, ASD and ASD+Rem groups, and each group included three independent samples from different mice. DESeq2 was used to perform differential gene expression analysis, and the thresholds for identifying differentially expressed genes were set at |log_2_Fold Change| ≥ 1 and p‐value < 0.05. OE Cloud tools (https://cloud.oebiotech.com) were used for data analyses including Gene Set Enrichment Analysis (GSEA).

### 5.22 Immunofluorescence (IF) Staining

Mice were anesthetized and perfusion with 4% paraformaldehyde. The mice's brains were separated and preserved for 2 to 3 days in 4% paraformaldehyde. Once the brain has been fixed, use a sucrose gradient to dehydrate it. Find the matching VTA brain regions, submerge them in frozen OCT, then perform frozen slices with a 20 µm thickness after doing a simple PBS flush. Regarding cellular immunofluorescence staining, place the prepared polylysine‐coated slides in a 24‐well plate, inoculated MN9D cells into the 24‐well plate for culture, and add the culture medium containing the corresponding drug after they grow to an appropriate density. After 12 h of drug stimulation, the medium was removed and washed three times with PBS before fixation. Subsequent steps were consistent with tissue immunofluorescence. The tissue slices or cell climbing slices were fixed in 4% paraformaldehyde for 10 min, and then they were treated with a tissue permeabilization solution containing 0.3% Triton X‐100. Finally, an immunostaining blocking solution was used to block the samples for an hour. Primary antibodies, including rabbit monoclonal TH antibody (ab137869, abcam), mouse Ki67 antibody (9449S, CST), mouse GPX4 antibody (67763‐1‐Ig, proteintech), rabbit SLC7A11 antibody (26864‐1‐AP, proteintech), rabbit ACSL4 antibody (R24265, Zenbio) and rabbit TFR1 antibody (R381603, Zenbio) were added to the sections according to the experimental requirements. After incubation at 4°C overnight, the sections were placed at room temperature for 30 min and then rinsed with PBS. Then, the sections were incubated with the corresponding AF488 or AF594‐labeled fluorescent secondary antibody at 37°C in the dark for 1 h. The secondary antibodies specifically include Goat Anti‐Rabbit IgG H&L (Alexa Fluor 488) (ab150077, abcam), Goat Anti‐Rabbit IgG H&L (Alexa Fluor 594) (ab150080, abcam), Goat Anti‐Mouse IgG H&L (Alexa Fluor 594) (ab150116, abcam) and Goat Anti‐Mouse IgG H&L (Alexa Fluor 488) (ab150113, abcam). After being washed with PBS, the slides were mounted with an anti‐fluorescence quenching sealing agent containing DAPI. Then ImageJ software was used to calculate and analyze the ratio and mean fluorescence intensity of positive cells in the sections. The average fluorescence intensity was calculated as integrated density (IntDen) / area. Average fluorescence intensity unit: a.u. (arbitrary units).

### 5.23 Statistical Analysis

In this study, all data are presented as mean ± standard deviation, and GraphPad Prism 10.0 was used for analysis. Double‐blind testing procedures were used for behavioral assessments. The number of samples involved in statistical analysis in this study was all 6 per group. To examine more than two groups, one‐way analysis of variance (ANOVA) and two‐way ANOVA were employed, depending on the number of variables, and Tukey's multiple comparisons test was applied. *p* < 0.05 indicated a statistically significant difference. ^*^ *p* < 0.05, ^**^ *p* < 0.01, ^***^ *p* < 0.001, ^****^ *p* < 0.0001, and ns indicates no statistical difference.

## Author Contributions

Y.Z. conducted the experiments, analyzed the data, and drafted the manuscript. J.L. helped to analyze RNA sequence results and complete the animal, cell and WB experiments. C.T. participated in manuscript editing and funding acquisition. X.F. helped to design this study. M.S. assisted in animal experiments. Y.H. helped to conceive this study. K.Z. and J.Z. participated in experiment design, manuscript editing and funding acquisition. All authors read and approved the final manuscript.

## Conflicts of Interest

The authors declare no conflict of interest.

## Supporting information




**Supporting File 1**: advs74184‐sup‐0001‐SuppMat.doc.

**Supporting File 2**: advs74184‐sup‐0002‐DataFile.zip.

## Data Availability

The data that support the findings of this study are available from the corresponding author upon reasonable request.
