# Supplementary material for: Remimazolam Ameliorates Autistic‐Like Behaviors via Suppression of Ferroptosis in VTA Dopaminergic Neurons in a Mouse Model of ASD
Source: Adv Sci (Weinh). 2026 Feb 3;13(20):e08520. doi: 10.1002/advs.202508520 (PMC13067855; doi:10.1002/advs.202508520)
Supplement: Supplementary file 1 — Supporting File 1: advs74184‐sup‐0001‐SuppMat.doc. [file ADVS-13-e08520-s002.doc]

Supporting Information

**Remimazolam ameliorates autistic-like behaviors via suppression of ferroptosis in VTA dopaminergic neurons in a mouse model of ASD**

*Yuxin Zhang, Jianwei Lin, Chaoyang Tong, Xin Fu, Mengqin Shan, Yue Huang*, Kan Zhang*, Jijian Zheng**

**Figure S1**

**
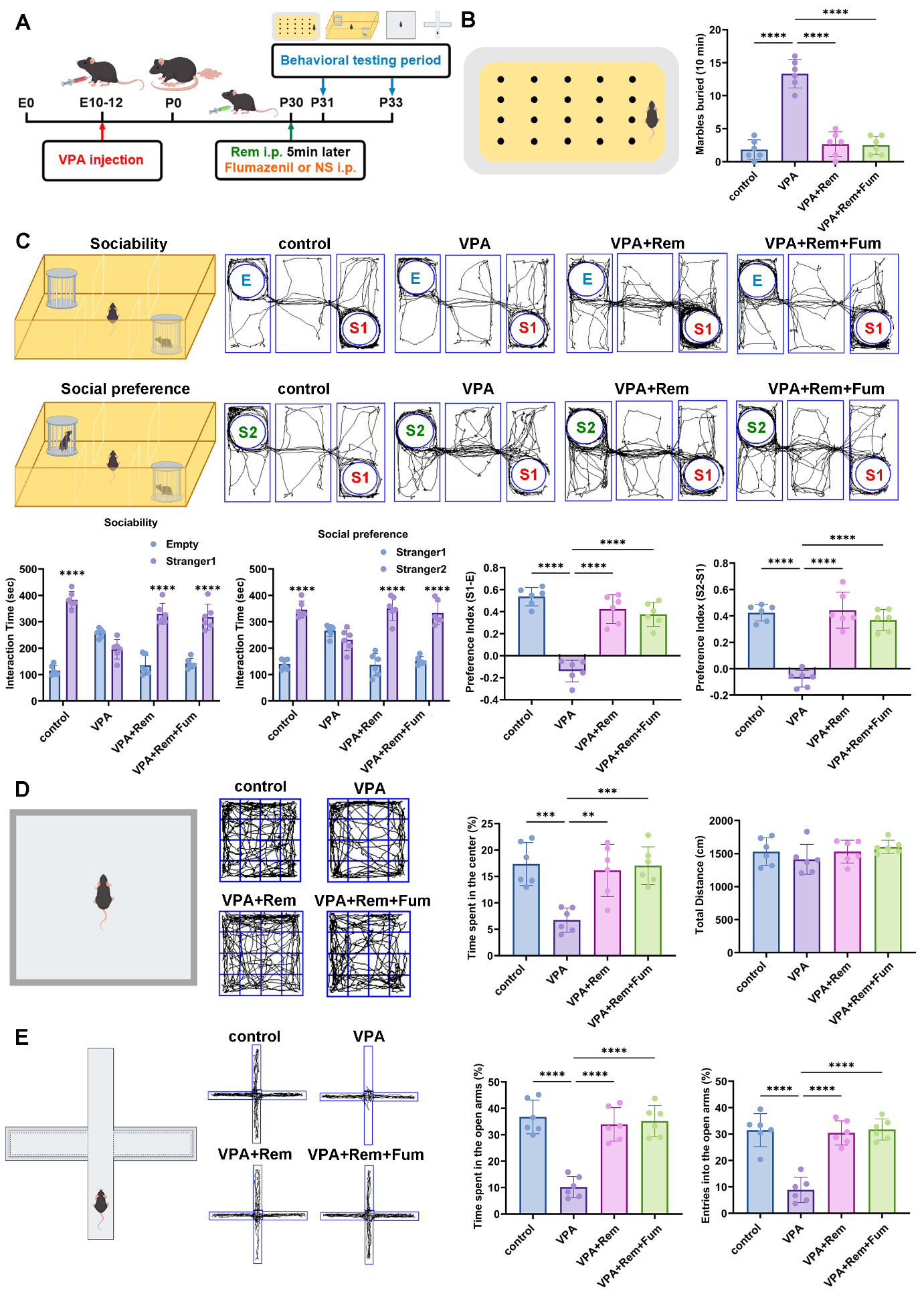
**

**Figure S1.** Flumazenil failed to reverse the therapeutic effects of remimazolam on autistic-like behaviors in VPA-exposed mice.

(A) An outline of the experimental process for administering drugs and performing behavior measures. (B) The schematic of marble burial experiment and the statistical graph of buried marbles number. *n* = 6 per group. (C) Representative traces of three-chamber social interaction test (E: Empty, S1: stranger 1, S2: stranger 2). Statistical graph of sociability and social novelty test. *n* = 6 per group (D) Representative traces of open-field test. Statistical graph of the percent of time spent in the center and total distance. *n* = 6 per group. (E) Representative traces of elevated plus maze test. Statistical graph of time and entries spent in open arms. *n* = 6 per group. Values was presented as mean ± SEM and analyzed by one-way ANOVA or Two-way ANOVA. ns > 0.05, **p* < 0.05, ***p* < 0.01, ****p* < 0.001 and *****p* < 0.0001.

**Figure S2**


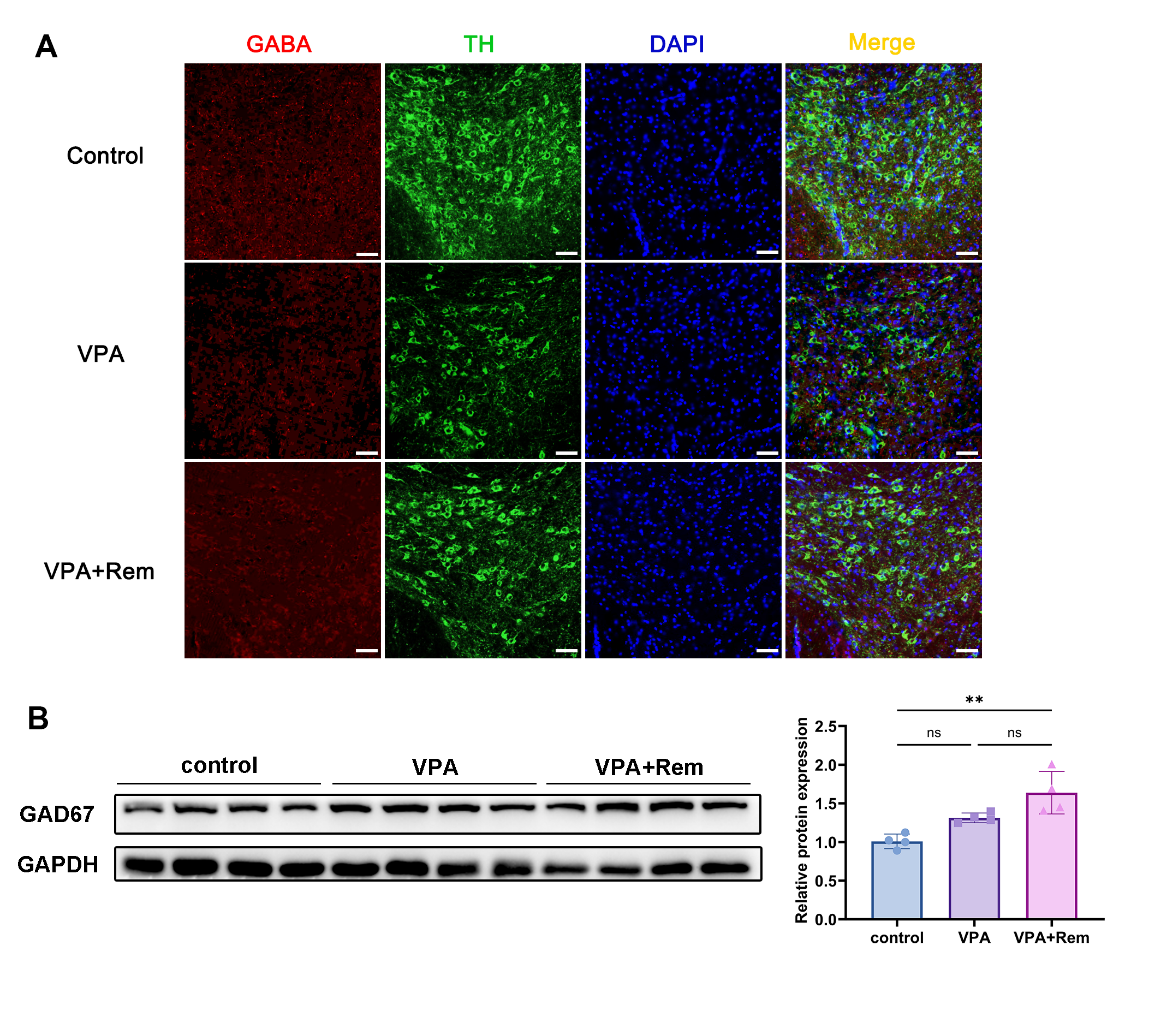


**Figure S2.** The results of GABA neurons in remimazolam treated VPA-exposed mice.

(A) Representative immunofluorescence images of GABA (red), TH (green) and DAPI (blue) in the VTA of VPA-exposed mice. Scale bar = 50μm (B) Representative protein bands of GAD67 and statistical graph of protein levels in the VTA of VPA-exposed mice. *n* = 4 per group. Values was presented as mean ± SEM and analyzed by one-way ANOVA. ns > 0.05 and ***p* < 0.01.

**Figure S3**

**
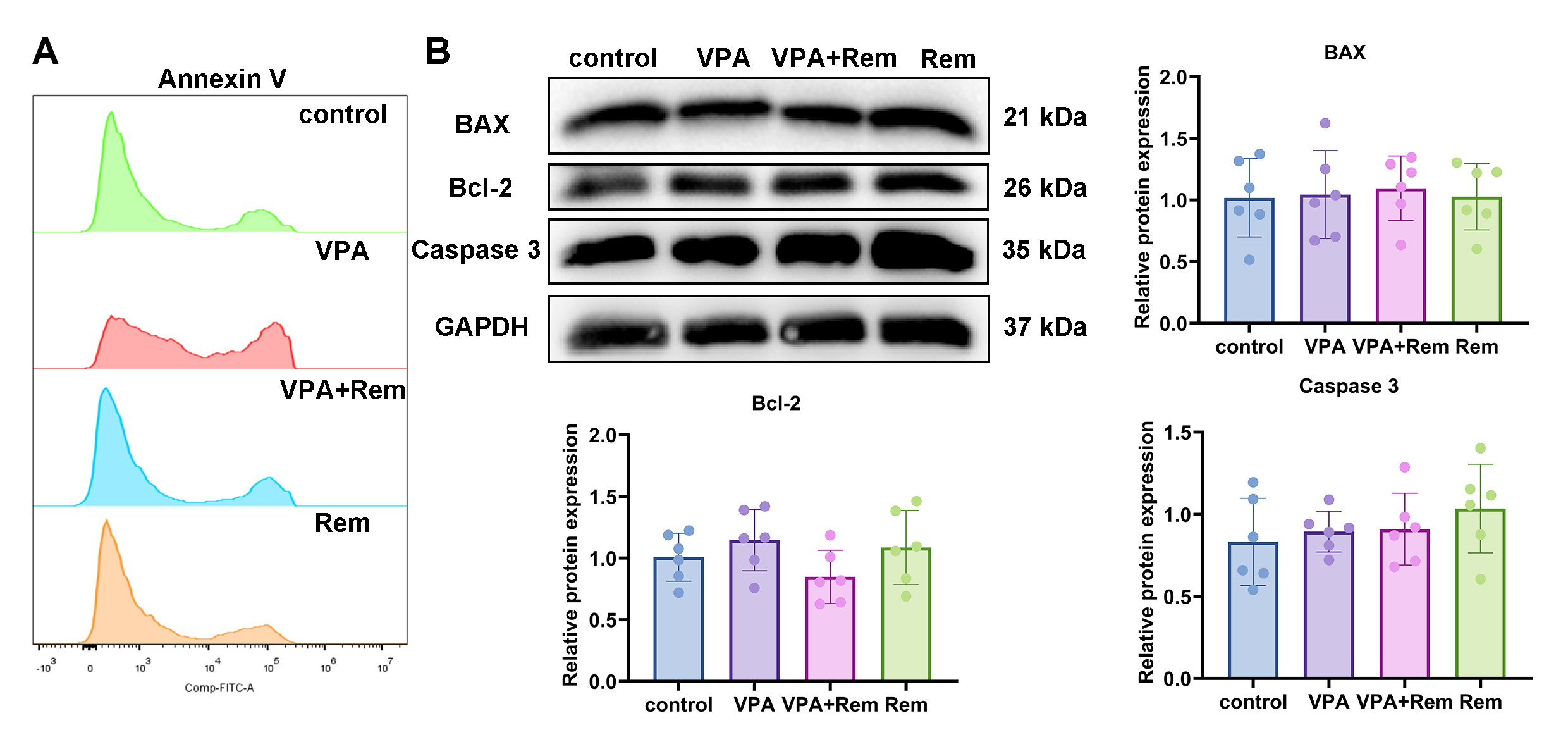
**

**Figure S3.** Remimazolam exerts a protective effect on VPA-exposed dopaminergic neurons independent of the apoptotic pathway.

1. Annexin V-FITC Apoptosis staining detected by Flow Cytometry. (B) Apoptosis related proteins (BAX, Bcl-2, and Caspase 3) levels in dopaminergic neurons. Values was presented as mean ± SEM and analyzed by one-way ANOVA.

**Figure** **S4**


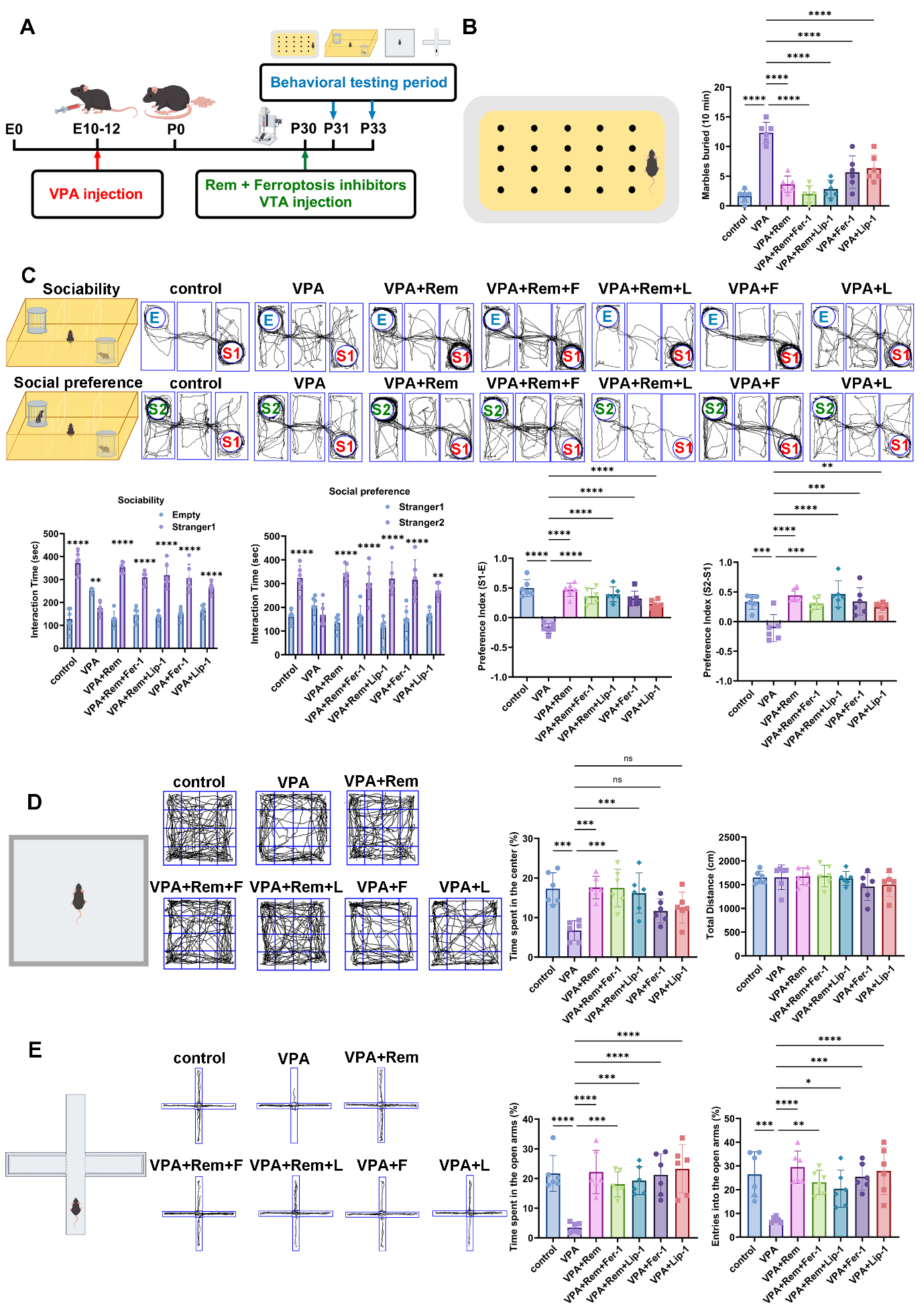


**Figure S4.** Ferroptosis inhibitors improve autistic-like behaviors in VPA-exposed mice.

(A) An outline of the experimental process for administering drugs and performing behavior measures. (B) The schematic of marble burial experiment and the statistical graph of buried marbles number. *n* = 6 per group. (C) Representative traces of three-chamber social interaction test (E: Empty, S1: stranger 1, S2: stranger 2). Statistical graph of sociability and social novelty test. *n* = 6 per group (D) Representative traces of open-field test. Statistical graph of the percent of time spent in the center and total distance. *n* = 6 per group. (E) Representative traces of elevated plus maze test. Statistical graph of time and entries spent in open arms. *n* = 6 per group. Values was presented as mean ± SEM and analyzed by one-way ANOVA or Two-way ANOVA. ns > 0.05, **p* < 0.05, ***p* < 0.01, ****p* < 0.001 and *****p* < 0.0001.

**Figure S5**

**
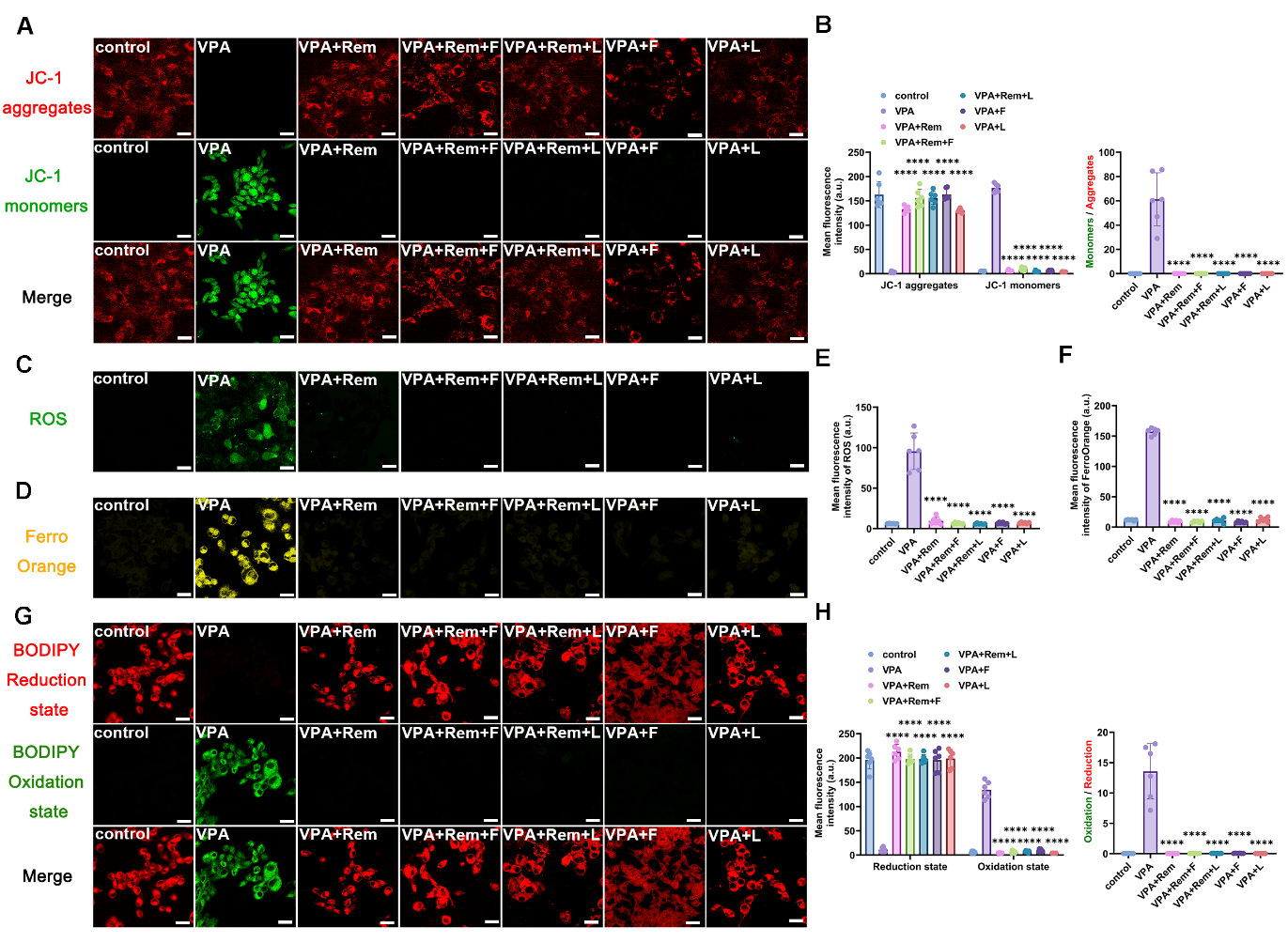
**

**Figure S5.** Ferroptosis inhibitors improve the characteristic changes of ferroptosis in VPA-exposed dopaminergic neurons.

(A) Representative immunofluorescence images of JC-1 monomers (green) and JC-1 aggregates (red) in MN9D dopaminergic neuron. Scale bar = 25μm. (B) Mean fluorescence intensity of JC-1 and ratio of monomers and aggregates. *n* = 6 per group. (C) Representative immunofluorescence images of ROS (green) in MN9D dopaminergic neuron. Scale bar = 25μm. (D) Representative immunofluorescence images of FerroOrange (orange) in MN9D dopaminergic neuron. Scale bar = 25μm. (E) Mean fluorescence intensity of ROS. *n* = 6 per group. (F) Mean fluorescence intensity of FerroOrange. *n* = 6 per group. (G) Representative immunofluorescence images of BODIPY Oxidation state (green) and BODIPY Reduction state (red) in MN9D dopaminergic neuron. Scale bar = 25μm. (H) Mean fluorescence intensity of BODIPY and ratio of Oxidation state and Reduction state. *n* = 6 per group. Values was presented as mean ± SEM and analyzed by one-way ANOVA or Two-way ANOVA. ns > 0.05, **p* < 0.05, ***p* < 0.01, ****p* < 0.001 and *****p* < 0.0001.

**Figure S6**

**
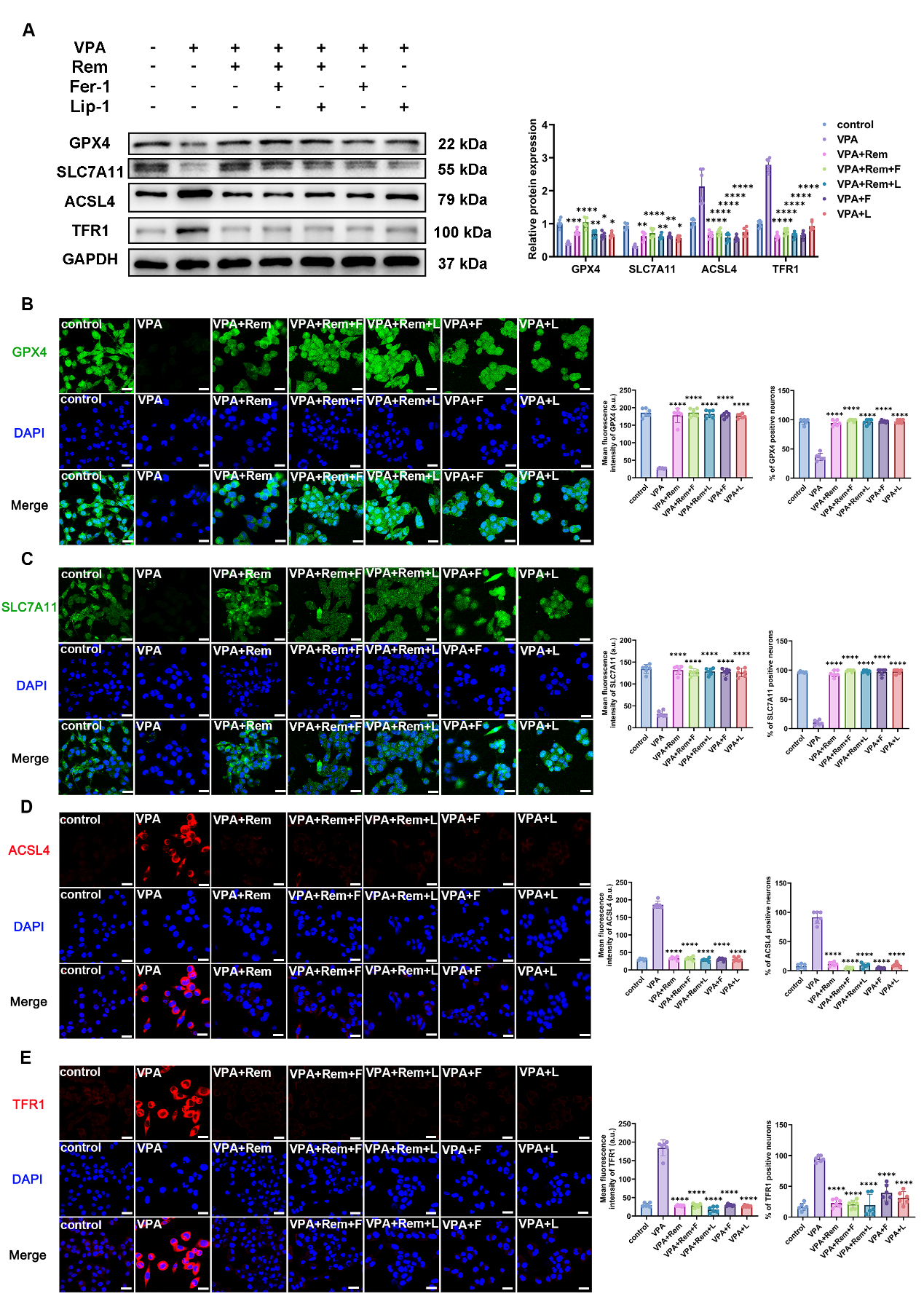
**

**Figure S6.** Ferroptosis inhibitors improve the key proteins of ferroptosis pathway in VPA-exposed dopaminergic neurons.

(A) Representative protein bands of GPX4, SLC7A11, ACSL4 and TFR1 in MN9D dopaminergic neuron. Statistical graph of protein levels of GPX4, SLC7A11, ACSL4 and TFR1. n = 6 per group. (B) Representative immunofluorescence images of GPX4 (green) and DAPI (blue) in MN9D dopaminergic neuron. Statistical graph of mean fluorescence intensity and positive neurons of GPX4. n = 6 per group. Scale bar = 25μm. (C) Representative immunofluorescence images of SLC7A11 (green) and DAPI (blue) in MN9D dopaminergic neuron. Statistical graph of mean fluorescence intensity and positive neurons of SLC7A11. n = 6 per group. Scale bar = 25μm. (D) Representative immunofluorescence images of ACSL4 (red) and DAPI (blue) in MN9D dopaminergic neuron. Statistical graph of mean fluorescence intensity and positive neurons of ACSL4. n = 6 per group. Scale bar = 25μm. (E) Representative immunofluorescence images of TFR1 (red) and DAPI (blue) in MN9D dopaminergic neuron. Scale bar = 25μm. Statistical graph of mean fluorescence intensity and positive neurons of TFR1. n = 6 per group. Values was presented as mean ± SEM and analyzed by one-way ANOVA or Two-way ANOVA. ns > 0.05, **p* < 0.05, ***p* < 0.01, ****p* < 0.001 and *****p* < 0.0001.
