# Supplementary figures and images for: Remimazolam Ameliorates Autistic‐Like Behaviors via Suppression of Ferroptosis in VTA Dopaminergic Neurons in a Mouse Model of ASD
Source: Adv Sci (Weinh). 2026 Feb 3;13(20):e08520. doi: 10.1002/advs.202508520 (PMC13067855; doi:10.1002/advs.202508520)

Original Blots


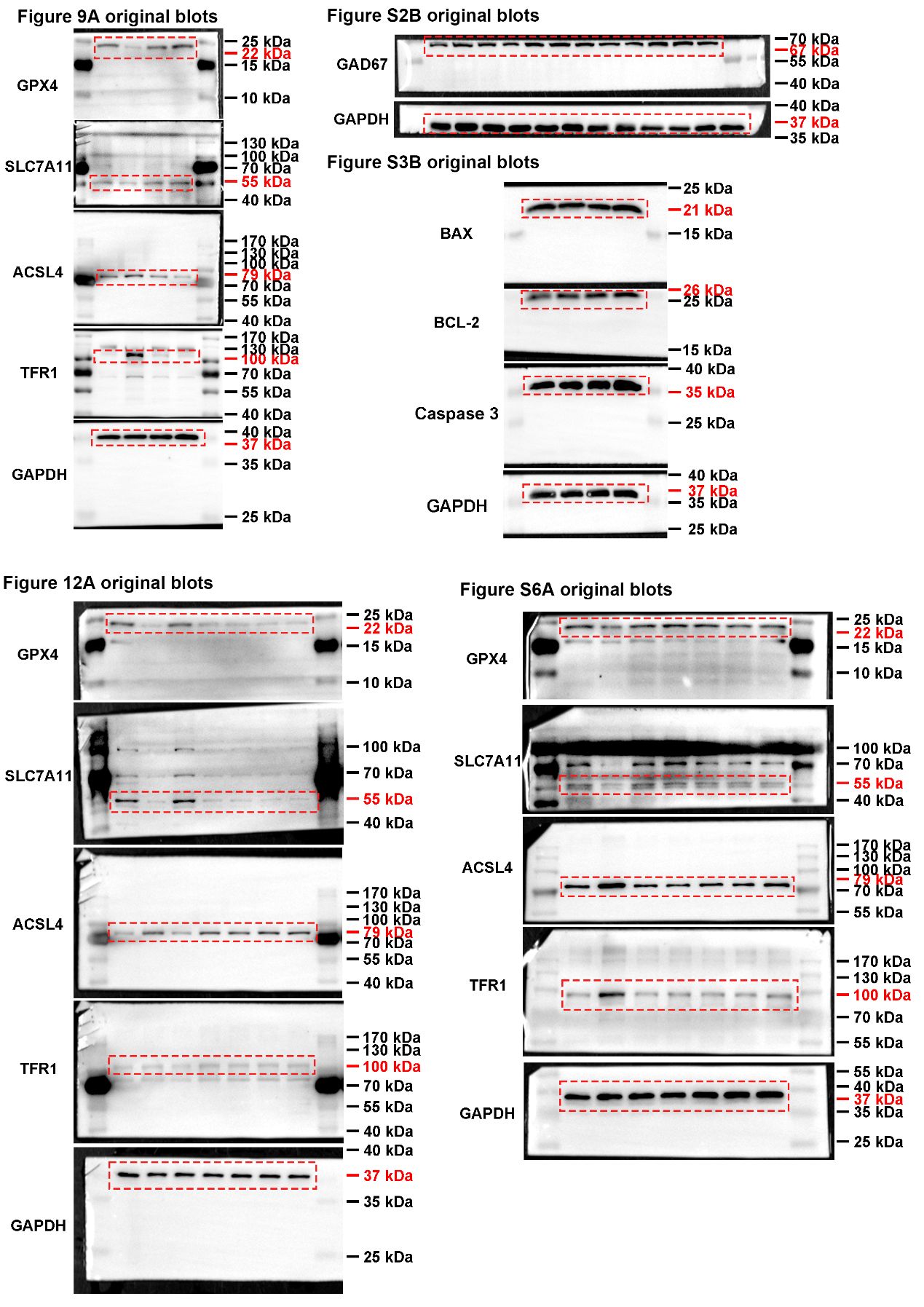

Supplement: Supplementary file 2 — Supporting File 2: advs74184‐sup‐0002‐DataFile.zip. [file ADVS-13-e08520-s001.zip › Original blots.docx]
